# An optimised CRISPR/Cas9 protocol to create targeted mutations in homoeologous genes and an efficient genotyping protocol to identify edited events in wheat

**DOI:** 10.1186/s13007-019-0500-2

**Published:** 2019-10-24

**Authors:** Xiucheng Cui, Margaret Balcerzak, Johann Schernthaner, Vivijan Babic, Raju Datla, Elizabeth K. Brauer, Natalie Labbé, Rajagopal Subramaniam, Thérèse Ouellet

**Affiliations:** 1 Ottawa Research and Development Centre, 960 Carling Avenue, Ottawa, ON K1A 0C6 Canada; 2 Department of Biology, University of Ottawa, 75 Laurier Ave E, Ottawa, ON K1N 6N5 Canada; 3 Aquatic and Crop Resource Development, National Research Council Canada, 110 Gymnasium Place, Saskatoon, SK S7N 0W9 Canada

**Keywords:** CRISPR/Cas9, Wheat, Gene editing in homoeologous genes, sgRNA, Transgenic plants, Genotyping

## Abstract

**Background:**

Targeted genome editing using the Clustered Regularly Interspaced Short Palindromic Repeats (CRISPR)/Cas9 system has been applied in a large number of plant species. Using a gene-specific single guide RNA (sgRNA) and the CRISPR/Cas9 system, small editing events such as deletions of few bases can be obtained. However larger deletions are required for some applications. In addition, identification and characterization of edited events can be challenging in plants with complex genomes, such as wheat.

**Results:**

In this study, we used the CRISPR/Cas9 system and developed a protocol that yielded high number of large deletions employing a pair of co-expressed sgRNA to target the same gene. The protocol was validated by targeting three genes, *TaABCC6*, *TaNFXL1* and *TansLTP9.4* in a wheat protoplast assay. Deletions of sequences located between the two sgRNA in each gene were the most frequent editing events observed for two of the three genes. A comparative assessment of editing frequencies between a codon-optimized Cas9 for expression in algae, crCas9, and a plant codon-optimized Cas9, pcoCas9, showed more consistent results with the vector expressing pcoCas9. Editing of *TaNFXL1* by co-expression of sgRNA pair was investigated in transgenic wheat plants. Given the ploidy of bread wheat, a rapid, robust and inexpensive genotyping protocol was also adapted for hexaploid genomes and shown to be a useful tool to identify homoeolog-specific editing events in wheat.

**Conclusions:**

Co-expressed pairs of sgRNA targeting single genes in conjunction with the CRISPR/Cas9 system produced large deletions in wheat. In addition, a genotyping protocol to identify editing events in homoeologs of *TaNFXL1* was successfully adapted.

## Background

The type II prokaryotic Clustered Regularly Interspaced Short Palindromic Repeats (CRISPR)/CRISPR-associated (Cas) system was initially identified in 2007 [1], and the unique features of this system have been explored widely in both Eubacteria and Archaea [2]. The first application of CRISPR as a gene editing tool was reported in human and mouse cells, demonstrating that Cas9 nucleases could induce precise cleavage at targeted genome loci with the presence of short RNA guiding sequences [3, 4]. In plants, this technology has been shown to have a high potential for gene editing both in monocot and dicot plants [5]. To date, several genes targeted in model plants such as *Arabidopsis thaliana* and tobacco (*Nicotiana tabacum*) have been successfully edited using this technology [6]. Also, this technology has been successfully applied for gene editing in several crops including rice (*Oryza sativa*), maize (*Zea mays*) and sorghum (*Sorghum bicolor*) genomes [6–8].

Bread wheat (*Triticum aestivum*) plays a central role in global food and feed crop consumption and is one of the most widely cultivated crops around the world. The wheat genome comprises three subgenomes (A, B and D), each representing a set of seven chromosomes. The size of the wheat genome is approximately 16 Gb; over 80% of it is composed of highly repetitive sequences and transposable elements and estimated to encode 108,000 high-confidence protein-coding loci [9].

Editing events obtained in wheat using CRISPR/Cas9 and one single guide RNA (sgRNA) are often associated with single nucleotide deletion/addition or small deletions [10–13]. However loss-of-function mutants with larger deletion, preferably in most of the homoeologs for a given gene, are desired to observe a phenotypic change in wheat., The use of paired sgRNA for CRISPR/Cas9 editing of a targeted gene has been shown to result in larger deletions in *Arabidopsis thaliana*, rice and kiwifruit [14–16]; however this method has not been tested in wheat. Here, we explore this approach, and present a protocol for targeted deletion of gene fragments using paired sgRNA and the CRISPR/Cas9 system in wheat and validate it for three wheat genes in a wheat protoplast system: an ABC transporter (*TaABCC6*), a lipid transfer protein (*TansLTP9.4*) and a putative transcription repressor named *TaNFXL1*. These three genes were previously identified to be associated with susceptibility (*TaABCC6, TaNFXL1)* and resistance (*TansLTP9.4*) to Fusarium head blight (FHB), a devastating fungal disease of wheat [17, 18]. In addition, a direct comparison of editing efficiency in wheat protoplasts was performed with two modified nucleases Cas9, crCas9 and pcoCas9, both derived from the *Streptococcus pyogenes* Cas9 [6, 19].

The utility of paired sgRNA-based approach for targeted editing of homoeologous genes has been tested in transgenic wheat plants for *TaNFXL1*. Identifying specific editing events in transgenic wheat plants is challenging due to the high homology of genes among the three subgenomes and the number of duplication events for those genes [9]. Sequencing of homoeolog-specific amplicons is most often used for this. Here we present a rapid, inexpensive screening method for edited genes in transgenic wheat plants. This method, adapted from a single-tube, nested PCR method using two sequence specific primers and a universal fluorescent-labeled primer [20], exploits single nucleotide polymorphisms (SNP) present in *TaNFXL1* homoeologs.

## Results

### sgRNA design and vector construction

Three genes of interest, herein referred to as *TaABCC6*, *TansLTP9.4* and *TaNFXL1*, were selected for evaluating targeted gene editing. For each gene, two sgRNA were designed, targeting the conserved homoeologous sequences in wheat subgenomes (Table 1). The sgRNA were designed using the genomic sequence information available in the Wheat Sequence Survey V2 [21], combined with expressed sequence tags (EST) available in a local database of assembled public wheat ESTs [22] and amplicon sequencing obtained from the spring wheat cultivar Fielder (unpublished observations). Using the wheat genomic sequence RefSeq v1.0 [9] two closely related genes on each of the three subgenomes were identified for *TaABCC6* and *TaNFXL1*, while *TansLTP9.4* was encoded by a single gene per subgenome. Additional file 1 provides the list of homoeologous genes, together with the sequences best matching to each sgRNA. The efficacy of the designed sgRNA to guide Cas9 to specifically cut the target sequence was tested using an in vitro assay [23]. As shown in Additional file 2, two smaller DNA fragments with expected sizes were clearly observed after cleavage with each sgRNA. Each pair of sgRNA targeting the same gene was cloned into a single expression vector, together with one of two Cas9 (see below), as illustrated in Additional file 3 and described in “Methods”.

**Table 1:** Selected sgRNA for *TaABCC6, TansLTP9.4* and *TaNFXL1*

sgRNA	Sequence (5′-3′)
ABCC6-sgRNA-1	CACGCCGTCGAGATTACTGG
ABCC6-sgRNA-2	AGTACTCACGGAGATCCAAG
nsLTP9.4-sgRNA-1	GCCGTGCGTGGCGTACGTGA
nsLTP9.4-sgRNA-2	AGTGCTGCTCCGGCGTGCAG
NFXL1-sgRNA-1	TGACTGGCACAACGCAAGGT
NFXL1-sgRNA-2	GATGGAGTTGGTGTGCCGCA

### Assessment of editing frequency for three pairs of co-expressed sgRNA

Wheat protoplasts isolation and transformation procedures using the cultivar Roblin routinely yielded around 60% transfection efficiency (Additional file 4), similar to the frequencies obtained by Shan et al for protoplasts from shoot tissues of the wheat cultivar Bobwhite [24]. The protoplast system was used to determine the editing frequency and specificity of the selected sgRNA as well as to compare the differences in editing between two modified version of the Cas9 isolated from *S. pyogenes*, one that is codon-optimized for expression in the algae *Chlamydomonas reinhardtii* (referred to as crCas9) [6] and one that is codon-optimized for expression in plants (called pcoCas9) [19].

To estimate the editing frequency for each gene, genomic DNA from protoplasts were isolated from three independent transformation experiments (Additional file 5) and were used to amplify a fragment of each target gene that included both sgRNA target sites (Additional file 6). High throughput sequencing (HTS) of the amplicons was performed to quantify targeted mutations by crCas9 on all three target genes (samples ABCC6-1 to -5, nsLTP9.4-1 to -5 and NFXL1-1 to -5) and by pcoCas9 on *TaNFXL1* (samples pcoNFXL1-1 to -5). Overall, more than 90% of the reads were successfully mapped to the reference sequences, except for samples ABCC6-1 to -5 where about only 80% of the reads were mapped to the reference sequences (unpublished observations). Although the ABC transporters constitute a large gene family in wheat, no reads mapped to other ABC transporter genes than the six ABCC6 homoeologs. Analysis showed that editing frequency of *TaABCC6* was consistent between transformation events while for *TansLTP9.4* and particularly *TaNFXL1*, editing frequency varied from 0% (no editing) up to 42% (Table 2). The variations observed could be in part associated with the batch of isolated protoplasts (Additional file 5). Total editing frequency was also compared between the two modified nucleases crCas9 and pcoCas9. As shown, the editing frequencies in the samples transformed with the vector expressing pcoCas9 were more consistent (coefficient of variation, CV = 38%) than those transformed with the vector expressing crCas9 (CV = 87%); however, none of the *TaNFXL1* samples edited by pcoCas9 reached the high level of editing observed with crCas9 (42.2%) (Table 2).

**Table 2:** Estimated total editing frequency for *TaABCC6, TansLTP9.4* and *TaNFXL1*

Sample	Editing frequency^a^	Sample	Editing frequency	Sample	Editing frequency	Sample	Editing frequency
ABCC6-1	9.3	nsLTP9.4-1	9.1	NFXL1-1	8.4	pcoNFXL1-1^b^	10.2
ABCC6-2	6.6	nsLTP9.4-2	1.9	NFXL1-2	18.3	pcoNFXL1-2	8.6
ABCC6-3	9.1	nsLTP9.4-3	11.3	NFXL1-3	42.2	pcoNFXL1-3	15.0
ABCC6-4	13.0	nsLTP9.4-4	11.9	NFXL1-4	22.8	pcoNFXL1-4	21.2
ABCC6-5	9.0	nsLTP9.4-5	0.0^c^	NFXL1-5	0.0^c^	pcoNFXL1-5	20.7

^a^Editing frequency (%) = (reads with modification/(mapped reads + reads with modification)) × 100

^b^pcoCas9 was used instead of crCas9 for the samples pcoNFXL1-1 to 5

^c^No modification was detected in those samples

**Fig. 1:**
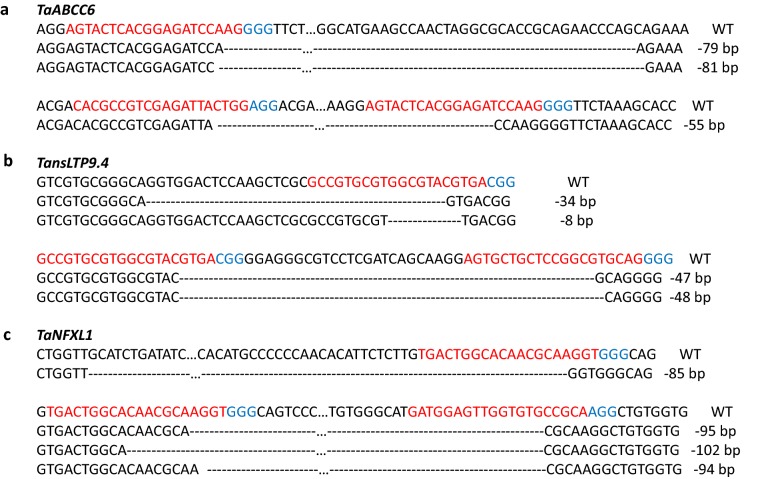
Examples of editing identified for each of the three targeted genes. Targeted deletions observed for *TaABCC6* (**a**), *TansLTP9.4* (**b**), and *TaNFXL1* (**c**). For each gene, the 20 nt sgRNA sequences are in red while the PAM structures are marked in blue. WT: Fielder sequence

The major type of editing observed in the transformed protoplasts was deletion of fragments larger than 40 bp. Representative examples are presented in Fig. 1 and the complete list of editing observed is detailed in Additional file 7, sections A to F. A higher percentage (five to sixfold) of deletions was obtained for the regions flanked by the two sgRNA, especially for the genes *TaABCC6* and *TansLTP9.4*, where the distance between the sgRNA target sites was smaller (Additional files 6, 7E). In addition to deletions, sequence insertion was observed only for *TaNFXL1* samples, and with high frequency, when exposed to both versions on the Cas9 nuclease (Additional file 7F). After taking into account total editing frequency, we did not observe significant differences in frequency in any of the modification types between the two Cas9 (crCas9 and pcoCas9) nucleases.

The HTS data from the transformed protoplasts also allowed the quantification of editing events targeted by each sgRNA (Additional file 8). The editing efficiencies for sgRNA-1 and -2 were very consistent between independent transformation events. In samples ABCC6-1 to -5 and nsLTP9.4-1 to -4, similar frequencies of editing were observed for sgRNA-1 and -2. However, there was a higher editing efficiency at the NFXL1-sgRNA-2 site than at the NFXL1-sgRNA-1 site in samples NFXL1-1 to -4 and pcoNFXL1-1 to -5. The differences in editing efficiency between these two sgRNA may have contributed to the infrequent occurrence of deletion of the fragment located between the two sgRNA in *TaNFXL1* samples, observed in only one of 10 samples (Additional file 7E). Differences in total editing frequencies for *TaNFXL1* between crCas9 and pcoCas9 were reflected in the results with individual sgRNA (Additional file 8).

With the recent availability of the wheat genomic sequence RefSeq v1.0 [9], the HTS amplicon data was reanalysed to identify the reads associated with each homoeolog of *TaABCC6* and *TaNFXL1*, using the samples with the highest total editing frequency (ABCC6-4, NFXL1-3, pcoNFXL1-4) as well as a control sample from non-edited Fielder protoplasts. For both *TaABCC6* and *TaNFXL1*, two of the homoeologs with a perfect match to sgRNA-1 and sgRNA-2 were represented in the HTS data (Table 3A, Additional file 1). The total number of reads that could be mapped with confidence to each of these homoeolog varied by three to 15-fold, indicating differences in efficiency of amplification between homoeologs; however, editing efficiency could be measured for each of those homoeologs. Fairly consistent editing frequency was observed between the two homoeologs of *TaABCC6* and *TaNFXL1* (≤ 2-fold difference), especially when crCas9 was used (Table 3A).

**Table 3:** Evaluation of editing accuracy for *TaABCC6* and *TaNFXL1* using homoeologs with perfect match (A) or mismatched bases (B) to the sgRNA

(A)					
Gene	On-target gene ID^a^	Total mapped reads^b^	Reads with modifications	Editing frequency^c^	
*TaABCC6*	TraesCS2A01G451300	14,718	1059	7.2	
	TraesCS2D01G451100	5337	235	4.4	
*TaNFXL1* (edited by crCas9)	TraesCS7A01G518800	1780	294	16.50	
	TraesCS7B01G434700	26,991	4142	15.30	
*TaNFXL1* (edited by pcoCas9)	TraesCS7A01G518800	3479	357	10.3	
	TraesCS7B01G434700	22,519	1062	4.7	

(B)					
Gene	Off-target Gene ID^a^	Total mapped reads^b^		Reads with modifications	Editing frequency^c^
		Edited by crCas9	Edited by pcoCas9		
*TaABCC6*	TraesCS2B01G472800	15205	N/A	252	1.7
	TraesCS2A01G451500	1722	N/A	0	< 0.06
	TraesCS2D01G451300	1987	N/A	0	< 0.05
*TaNFXL1*	TraesCS7D01G688900LC	2621	2887	0	< 0.04

*N/A* not applicable

^a^Only genes for which a specific fragment was PCR-amplified and sequenced are presented here. See Additional file 1 for more details

^b^The number of reads mapping perfectly to the corresponding target or off-target sequence

^c^Editing frequency (%) = (reads with modification/(mapped reads + reads with modification)) × 100

The HTS amplicon data also contained sequences for three (*TaABCC6*) and one (*TaNFXL1*) homoeologs which have target sites containing mismatches with the sgRNA (Table 3B, Additional file 1). No editing was detected in the *TaABCC6* homoeolog with 2 mismatches to sgRNA-1 and 1 to sgRNA-2 nor the *TaNFXL1* homoeolog with 3 mismatches to sgRNA-1, suggesting that editing was below 0.6%. Of the two *TaABCC6* homoeolog with only one mismatched base to one or both sgRNA, TraesCS2B01G472800 showed a notable level of editing, only 2.6- to 4-fold lower than for homoeologs with perfect match to the sgRNA, while no editing was detected for TraesCS2D01G451300 even though sgRNA-2 had a perfect match to it.

### Editing of *TaNFXL1 in* transgenic plants using a co-expressed pair of sgRNA

Transient silencing experiments indicated that reduced expression of the gene *TaNFXL1* was associated with reduced susceptibility to FHB of wheat [17, 18]. To confirm those results, CRISPR editing of *TaNFXL1* was performed *in planta.* For that purpose, a cassette containing the crCas9 coding sequence as well as the gBlock pair containing the two sgRNA was assembled in a binary vector for wheat transformation; the cloning strategy as well as the final transformation vector are presented in Additional file 9 and described in “Methods”. Progeny from four transgenic plants expressing crCas9 and the two sgRNA were characterized for editing events.

**Fig. 2:**
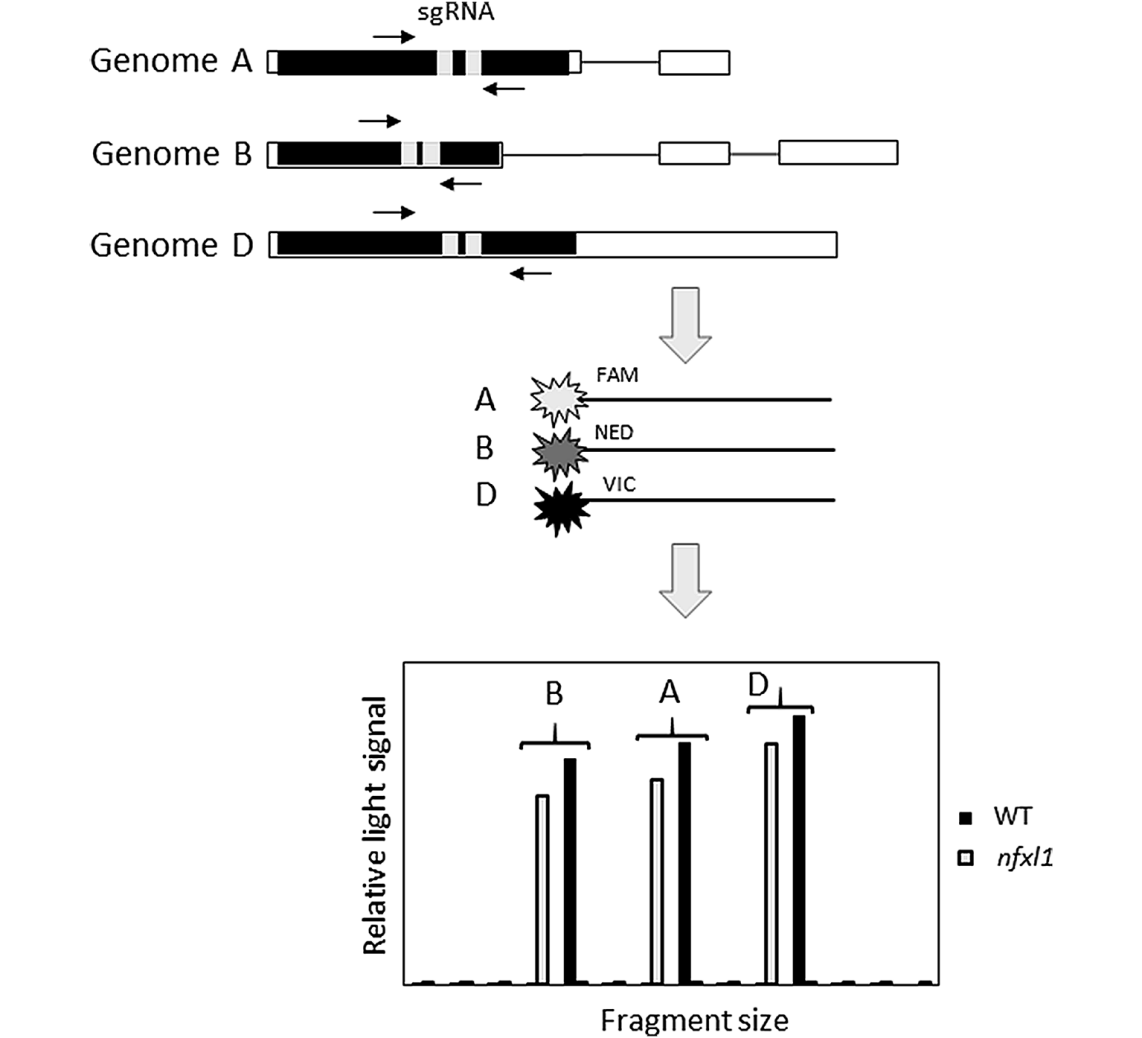
Schematic representation of the genotyping protocol to detect CRISPR editing events in wheat genes. Rows starting with Genome A, B and D illustrate the three homoeologous genes with the best match to *TaNFXL1*, with black and white boxes representing coding and non-coding exons respectively, horizontal lines introns and light grey boxes sgRNA positions. Horizontal arrows indicate the position of the homoeolog-specific PCR primers used for the first round of PCR. FAM, NED and VIC fluorescent dyes were used in a second PCR amplification to label the amplicons from the homoeologs on subgenomes A, B and D respectively. The bottom panel is a schematic representation of an electropherogram depicting possible results for non-edited (WT) and CRISPR-edited (*nfxl1*) homoeologs from subgenomes A, B and D

A genotyping protocol adapted from Schuelke et al [20] was designed to identify editing events in any of the 6 homoeolog genes for *TaNFXL1*; a schema of the two-step protocol is presented in Fig. 2 and details are provided in “Methods”. Briefly, SNP between the homoeologs were exploited to amplify homoeolog-specific gene fragments that included both sgRNA target sites; then fragments from each of two groups (gene X and Y groups) of three homoeologs were individually labeled in a second PCR amplification using one gene-specific primer and a universal primer labelled with one of three fluorescent dyes (FAM, NED and VIC), combined and separated by capillary electrophoresis along a size standard, providing size measurement of the PCR amplicons and thereby determination of the size of the deletion (insertion) in each edited homoeolog. This method was much cheaper than HTS for genotyping of a large number of progeny due to the reduced costs of separation by capillary electrophoresis rather than sequencing and to the use of labeled universal primers rather than a labeled, gene-specific primer.

A nomenclature was developed to report the complex editing events observed in *TaNFXL1* homoeologs in the first generation (T1) from transgenic plants expressing Cas9 and the two sgRNA, with the wild type genotype of Fielder being described as AXXYY BXXYY DXXYY. X and Y represent the *TaNFXL1* genes from groups X and Y on each of the three subgenomes A, B and D (Additional file 1); lower case x and y indicate edited alleles and subscript numbers distinguish between different editing events in those alleles (Table 4, Additional file 10).

**Table 4:** Number of T1 progeny with indicated genotypes from four transgenic wheat plants that contained Cas9 and sgRNA pairs for *TaNFXL1*

Genotype	Number of T1 progeny from plant:			
	NFXL1_1	NFXL1_2	NFXL1_3	NFXL1_4
AXXYY BXXYY DXXYY^a^	3	9	5	13
Ax_2_x_2_y_1_y_1_ Bx_2_x_2_y_2_y_2_ Dx_1_x_2_Yy_2_	6			
Ax_2_x_2_y_1_y_1_ Bx_2_x_2_y_2_y_2_ Dx_2_x_2_Yy_2_	4			
Ax_1_x_2_y_1_y_1_ Bx_1_x_1_y_1_y_2_ Dx_2_x_3_Yy_1_y_2_		2		
AXx_3_YY BXx_4_YY DXXYY			2	
AXx_3_YY BXXYY DXXYY			2	
AXXYy_7_ BXXYY DXXYY				5
Other individual unique patterns	8	7	15	2
Incomplete	2	3	0	4

^a^Genotype code: A, B and D: wheat subgenomes; X: the two alleles for the gene X group in each subgenome, with X as the wild type allele in Fielder and x_n_ for an allele with a distinct editing n; Y and y: alleles for the gene Y group. See Additional file 10 for complete genotyping data

We observed editing events at a frequency of 87, 52, 79 and 29%, respectively, in the T1 progeny from plants NFXL1_1, NFXL1_2, NFXL1_3 and NFXL1_4 (Table 4, Additional file 10). Editing of *TaNFXL1* was particularly frequent in progeny of NFXL1_1 and NFXL1_2; at least 18 out of 23 progeny of NFXL1_1 showed editing in one or both alleles of every homoeolog, while 11 out of 21 progeny of NFXL1_2 were edited in one or both alleles of one to six homoeologs. Mono-allelic editing events were mostly observed in progeny from NFXL1_-3 and -NFXL1_4. There was a large number of progeny harboring unique editing patterns while some editing patterns were common to 2 to 6 progeny from the same transgenic plant. A few progeny from NFXL1_2 and NFXL1_3 had three genotyping amplicons for a given homoeolog (eg gene DY in NFXL1_2_5, gene BX in NFXL1_3_2), suggesting the presence of chimeric tissues. In progeny of NFXL1_1 and NFXL1_2, the most frequent deletion in five of the 6 homoeologs was a deletion of about 95 bp, the same size as the distance between the two sgRNA, while most other deletions were between 3 and 54 bp and most likely associated with editing at only one of the two sgRNA targets.

## Discussion

Targeted gene editing is increasingly being used in plants to generate diverse gene variants, including loss of function alleles for defining functionalities, and also to remove deleterious alleles in crops. However the use of single gRNA to edit the gene of interest primarily produces small deletions and do not necessarily result in null alleles. In this study, we have developed and investigated the use of pairs of co-expressed sgRNA targeting a single gene using the CRISPR/Cas9 system in wheat. A protoplast system was used to characterize the editing events. The key findings from these experiments showed that deletion of sequences between the two sgRNA occurred more frequently than any other type of deletions. Consistent with this, results from transgenic plants confirmed that deletion of the sequence between the two sgRNA in *TaNFXL1* was a frequent event. Taken together, these results demonstrate the usefulness of the approach with two sgRNA to produce larger deletions in targeted wheat genes.

The editing frequencies that we have observed were of the same order as the results obtained by Wang et al using co-expressed single sgRNA to simultaneously target four genes in a similar wheat protoplast system [25]. Variation in editing frequency between protoplast isolation batches, as observed in our experiments, have been noted before and high quality isolated protoplasts are considered to be a bottle-neck in CRISPR/Cas9 applications [26]. In our protoplast and *in planta* experiments, deletion of the fragment between the two sgRNA targeting the same gene was observed most frequently. Larger deletions associated with the use of paired sgRNA was also observed in the diploid species *Arabidopsis* and rice; in those two species, deleted fragments up to 459 bp and 170 kbp, respectively, were successfully obtained [14, 16]. Our results showed that similar editing events can be produced in wheat, including on multiple homoeologs. In the protoplast system, there was a large difference in frequency of deletion of the fragment located between the two sgRNA for *TaNFXL1* when compared to those for *TaABCC6*, *TansLTP9.4*; those fragments were respectively of 95, 56 and 47 bp. However that low frequency of edition of the large fragment was not observed in the transgenic plants edited in *TaNFXL1*. More experiments will need to be conducted to ascertain if the distance between the paired sgRNA affects the frequency of edition of large fragments. Similarly, additional investigation may clarify if a larger distance between the paired sgRNA contributes to a larger frequency of sequence insertion, as observed for *TaNFXL1*.

In the protoplast system, there was less variation in editing frequencies between samples when using pcoCas9 than when using crCas9. Those two Cas9 have not been compared in wheat before. A number of differences exist between the constructs expressing either Cas9. The crCas9 gene in the pCambia vector was driven by a 35S promoter, while pcoCas9 was under the control of a 35SPPDK promoter (constitutive 35S enhancer fused to the maize C4 pyruvate orthophosphate dikinase (C4PPDK) basal promoter) [6, 19]. The different promoters used in the two vectors may have affected Cas9 expression levels, leading to different editing efficiencies. In addition, an intron was inserted in the Cas9 gene during its original modification to pcoCas9 [19]; inclusion of such introns in a gene have been shown to increase mRNA accumulation and translation in transgenic plants [27].

In addition to editing events in the homoeologs with perfect match to both sgRNA, editing at lower frequency was observed in the protoplast system for one of the *TaABCC6* homoeolog with one base mismatch to each sgRNA while no editing was observed for the homoeolog with two mismatches to one of the sgRNA. This is consistent with the findings of Anderson et al [28], who showed in human cells that lower editing frequency was associated with sgRNA carrying one mismatch to the targeted sequence. Our results with transgenic plants confirm that editing from sequence with imperfect match to sgRNA can be observed in wheat and underscore the importance of avoiding the use of sgRNA for which up to three mismatch bases can be found in other parts of the genome. Now that a full sequence of the wheat genome for the cultivar Chinese Spring is available [9], it will be easier to design specific wheat sgRNA. Resequencing of amplicons for the targeted genes in the desired cultivar is recommended before the design of sgRNA, until genome sequence for a larger number of wheat cultivars becomes available.

Identification of specific editing events in plant species with a complex genome can present a significant challenge. In wheat, 55% of the genes have an homoeolog in each of the three subgenomes and 27% are present as tandem duplicates [9]. In our study, six homoeologs have been considered to characterize the editing events in *TaNFXL1* in the transgenic plants. Even though HTS is a very powerful technique that allows the identification of editing events in multiple genes when co-amplified, technical difficulties with amplification of fragments for sequencing with similar efficiency for all genes targeted and the high cost per sample of the procedure led us to adapt an alternate protocol for genotyping of a large number of progeny. The protocol was initially developed by Schuelke for genotyping populations with a large number of microsatellite markers [20]. The genotyping procedure presented here also present advantages on a screening method recently published [29] because it does not require the design and optimisation of gene-specific primers able to recognize the CRISPR target sequence only when not edited. Our results showed that the genotyping method presented here was a robust and powerful tool to characterize CRISPR/Cas9 gene editing events in T1 progeny of transgenic plants. The protocol will also be applicable to other plant species with a complex genome.

## Conclusions

In this study, we present a protocol to co-express pairs of sgRNA targeting the same gene using the CRISPR/Cas9 system and successfully validate its use to generate larger deletions in an optimized wheat protoplast system and in transgenic plants. In addition, we have developed a rapid and inexpensive genotyping protocol, allowing the identification of editing events in all homoeologs of a gene in complex genomes such as that of wheat. Integration of these two protocols will contribute to accelerating functional gene studies in wheat.

## Methods

### Design of sgRNA pairs

SgRNA were designed using an online program, sgRNA Designer [30] and each sgRNA received a score from 0 to 1, based on its predicted efficiency. For each gene, two sgRNA with high score and located within 100 bp of each other were selected. Specificity of sgRNA was further verified in wheat genomic sequence RefSeqv1.0 once it became available [31].

### In vitro test for individual sgRNA

For each gene, a genomic DNA fragment including the two selected sgRNA sites was amplified from the spring wheat cultivar Fielder; attention was paid to designing primers that generated a fragment producing asymmetry after the cleavage reaction (Additional file 6, Additional file 11). Those genomic fragments were amplified by PCR using the following reaction: 1× PfuTurbo Cx PCR buffer (Agilent, CA), 0.5 µM each of forward and reverse primers, 0.2 mM dNTPs, 50 ng of Fielder genomic DNA and 1.25 U of high-fidelity PfuTurbo Cx Hotstart DNA polymerase in a final volume of 25 µL. The PCR amplification protocol was set up as follows: incubation at 94 °C for 3 min, followed by 35 cycles using 94 °C for 30 s, 60 °C for 30 s and 72 °C for 1 min, and final elongation was at 72 °C for 10 min. PCR products were purified with PureLink^®^ Quick PCR Purification Kit (Invitrogen/Thermo Fisher Scientific, MA), following the manufacturer’s instructions.

The sgRNA in vitro transcription and in vitro digestion of purified PCR products with a Cas9 nuclease were performed using the ‘Guide-it Complete sgRNA Screening System’ kit (Clontech, Mountain View, CA), following the manufacturer’s instructions.

### Assembly and cloning of sgRNA pairs into an expression vector for expression in protoplasts

Before cloning into an expression vector, each sgRNA was assembled into a functional module referred to as a gBlock. Each gBlock included a wheat U6 promoter, a gene specific sgRNA sequence, and a sgRNA scaffold and a terminator (Additional file 12) [32]. All gBlocks were designed using Lasergene 10 (DNASTAR, Madison, WI) and synthetized by Integrated DNA Technologies (Coralville, IA) [33]. The two gBlocks targeting the same gene were assembled into a single cloning unit using Gibson assembly [34] as follows: 25 ng of each gBlock and 10 μL of Gibson Master Mix (New England Biolabs Ltd, Whitby, ON) in a 20-μL volume were incubated at 50 °C for 1 h. Primers with *Eco*RI and *Kpn*I restriction enzyme sites (Additional file 11) were used to amplify the assembled gBlock pairs by PCR using the PfuTurbo Cx Hotstart DNA Polymerase reaction and conditions described above. The assembly was confirmed on agarose gel followed by gel purification of the PCR products with the QIAquick Gel Extraction kit (Qiagen, Toronto, Canada).

About 50 ng of purified assembled gBlock pair was ligated with 2.5 U of T4 DNA ligase (Promega, WI) to 25 ng of linearized pJet1.2/blunt vector (Thermo Fisher Scientific), following the manufacturer’s instructions. The recombinant vectors were chemically transformed into TOP10 *Escherichia coli* competent cells (Thermo Fisher Scientific) and positive clones were confirmed by Sanger sequencing using the primers indicated in Additional file 11.

A modified pCambia 1302 vector containing a Cas9 nuclease, which originated from *S. pyogenes* and was codon-optimized for expression in *C. reinhardtii* (crCas9), was used [6]. The pCambia and pJet 1.2-sgRNA recombinant vectors were both digested with *Eco*RI and *Kpn*I (Thermo Fisher Scientific), according to manufacturer’s instructions. An additional vector expressing a plant codon-optimized version of spCas9, pFGC-pcoCas9, was a gift from Jen Sheen (Addgene plasmid # 52256 [19, 35]); it was used in combination with the pair of gBlocks for editing of *TaNFXL1*. In this case, a different reverse primer with an *Xma*I restriction enzyme site and the same Gib_assem_*Eco*RI-1F forward primer (Additional file 11) were used to amplify the assembled *TaNFXL1* gBlock pair in order to insert it into the pFGC-pcoCas9 vector. The digested vectors were gel purified as described above. Each digested, assembled gBlock pairs (21 ng) was ligated into the digested pCambia 1302 or pFGC-pcoCas9 vector (100 ng) using 3 U of T4 DNA Ligase (Promega, WI) as described above. Additional file 3 shows a schematic representation of the region of the modified pCambia 1302 vector containing Cas9 and the gBlock pair. Chemical transformation and Sanger sequencing verification were as described above. Large amounts of each expression plasmid were extracted from 100-mL *E. coli* cultures using NucleaBond Xtra Midi kit (Clontech), according to manufacturer’s instructions, and DNA final concentrations adjusted to 1000 ng/μL.

### Protoplast isolation and transformation to test sgRNA pairs editing efficiency

Protoplasts were prepared from fresh leaves of Fielder using a modified version of Shan et al [24] that was optimized for Fielder tissues. Briefly, seeds were sterilized with 75% ethanol for 1 min followed by 50% bleach (containing 8.25% sodium hypochlorite) for 10 min, then rinsed five times with sterile water. The plants were grown in sterilized Magenta™ boxes (W×L×H: 77 mm × 77 mm × 97 mm, Sigma-Aldrich, MO) containing MS medium (4.2 g/L Murashige and Skoog Salts, 10 g/L sucrose, 3 g/L phytagel, pH 5.8), in a growth chamber at 21 °C under 16 h-light/8 h-dark light cycle, with approximately 450 μmol m^−2^s^−1^ photosynthetic photon flux density for 12 days. Twenty to 25 leaves were harvested from 12-day-old seedlings, cut into thin strips (~1 mm), transferred to a Petri dish containing 12.5 mL enzyme solution [0.6 M mannitol, 10 mM CaCl_2_, 20 mM MES pH 5.8, 10 mM KCl, and freshly added 0.1% bovine serum albumin (BSA; Sigma-Aldrich, MO), 1.5% cellulose R10 (Yakult, Japan) and 0.75% macerozyme R10 (Yakult, Japan)], or sufficient amount to cover the leaf strips, vacuum infiltrated for 40 min in the dark and incubated as described in [24]. After tissue incubation, the liquid was gently poured through an EASYstrainer™ Cell Strainer (70 µm mesh size, Greiner Bio-One, NC) over a 50 mL Falcon centrifuge tube (Thermo Fisher Scientific). The Petri dish was rinsed twice with 20 mL of W5 solution (154 mM NaCl, 125 mM CaCl_2_, 2 mM MES pH 5.8, 5 mM KCl) and the liquid collected into the same 50 mL tube for centrifugation at 100*g* for 2 min at room temperature. The final protoplast pellet was gently resuspended in 10 mL of W5, kept on ice in the dark for at least 30 min; meanwhile cell density was determined. After one more centrifugation at 100*g* for 1 min, protoplast pellet was gently resuspended in MMG solution (0.4 M mannitol, 15 mM MgCl_2_, 4 mM MES pH 5.8) at a cell density of only 2.5 × 10^5^/mL.

For protoplast transformation, a modified version of [24] was followed, using a half-volume recipe with 10 μg of recombinant expression plasmids (1 μg/μL), 100 μL (~2.5 × 10^4^ cells) of protoplasts and 110 μL of freshly prepared PEG solution (40% polyethylene glycol (PEG, molecular weight = 4000), 200 mM mannitol, 100 mM CaCl_2_). Protoplasts were incubated in the dark for only 5 min before adding 440 μL of W5 and centrifuging at 100*g* for 2 min. The transformed protoplasts were resuspended in 2 mL of W5 and incubated as described in [24]. Two individual transformations were pooled for DNA extraction using “Illustra Nucleon Phytopure Genomic DNA Extraction Kit” (GE Healthcare Life Sciences, MA), following the manufacturer’s instructions.

Frequency of transformation was estimated for each batch of protoplasts using pMDC32-ZsGreen vector expressing a green fluorescent protein (ZsGreen) (Additional file 13). Vector pMDC32-ZsGreen was prepared by removing the attR1-ccdB-attR2 cassette from pMDC32 [36] (The Arabidopsis Information Resource) by digestion with *Xba*I and replacing it with a *Xba*I fragment containing the ORF of ZsGreen [37] from pZsGreen1-1 (Clontech). Transformed protoplasts were resuspended in only 200 μL of W5 and 10 μL of cell suspension was observed using an Axio Scope.A1 (Item No. 430035-9100-000; Carl Zeiss, USA) connected to a Colibri.2 light source (Carl Zeiss, USA). The protoplasts were observed 2 days after transformation at 200× magnification. For fluorescence microscopy, a 505 nm wavelength was selected on the light source while the filter No.3 was chosen on the microscope. The transformation frequency was estimated by calculating the ratio of the number of fluorescent cells counted in the dark field, to the total number of cells counted in the same but bright field. Photos were taken using a Canon EOS 60D camera.

### Quantification of gene editing in transformed protoplasts by high throughput sequencing

Genomic DNA isolated from transformed protoplasts as well as from untransformed protoplasts (control) were used to amplify gene fragments including the sgRNA sites. The *TansLTP9.4* DNA fragments were amplified with TraesCS5A01G147000-specific primers (Additional file 11), using CloneAmp™ HiFi PCR Premix (Clontech) as follows: 95 °C for 3 min, followed by 35 cycles of 98 °C for 10 s, 60 °C for 15 s and 72 °C for 10 s. Amplicons were purified as described above and sequenced by Analysis of Genome Evolution & Function (University of Toronto, Canada). Fragments for *TaABCC6* and *TaNFXL1* were amplified with primers designed from conserved regions, based on the sequence information publicly available at the time of design (Additional file 11). A two-round PCR amplification was performed using KOD Hot Start DNA Polymerase (Novagen, Canada) at 95 °C for 2 min, followed by 35 cycles of 95 °C for 20 s, 60 °C (1st round) or 65 °C (2nd round) for 10 s and 70 °C for 15 s. Gene-specific primers were used for the first round of amplification, PCR products purified and 10 ng of purified products used for the second round of amplification. Cocktails of modified forward and reverse primers at 20 µM each were used for that second step; for those, overhang adapter sequences as well as 0–3 “N” bases in between the adapter and the gene-specific sequences were added to the 5’ of the gene-specific primers (Additional file 11) to make PCR products compatible with the protocol used by the HTS service, Molecular Microbiology & Next Generation Sequencing Service (Ottawa Research and Development Centre, Canada). MiSeq systems (Illumina, USA) were used by each HTS service.

The HTS data was first analysed using CLC Genomics Workbench (version 10.0.1; Qiagen). Briefly, for each sample, sequencing quality was verified, 20 bases were removed at both 3′ and 5′ ends of each read to ensure removal of adapter sequences, then trimmed reads were paired with default settings. Trimmed, paired reads were then used to detect and quantify targeted mutations using the InDels and Structural Variants tools under Resequencing Analysis in the CLC Genomics Toolbox. “Create breakpoints” was selected under output options with settings set as: P-value threshold = 0.0001, maximum number of mismatch = 3, minimum quality score and minimum relative consensus sequence coverage = 0, and “Ignore broken pairs”. For each sample, four individual files were generated, including InDel, Structural Variants and Breakpoint analyses, and a report for Structural Variants. The results of InDel and Breakpoint analyses were exported in excel files and different types of deletions or insertions were mapped to the reference sequences manually. Editing frequency was calculated as (number of reads with modification divided by the sum of mapped reads + reads with modification) × 100.

A second analysis of the HST data was performed for three samples to look at gene editing in specific homoeologous genes. About 300 bp of sequences surrounding the sgRNA pairs for each of the six homoeologs of *TaABCC6* and *TaNFXL1* (Additional file 1) were retrieved from IWGSC Reference Sequence v1.0 [31]. To determine if each homoeolog was edited, some of the HTS data was reanalysed using the Cas-Analyzer tool [38, 39]. Settings were set at ‘use both ends’ for comparison range, minimum frequency 5, wild type marker 5; the aligned reads were then manually selected for analysis. Sequences from each homoeolog were used as the reference sequences for the analyses.

### Cloning of *TaNFXL1* sgRNA pair into an expression vector for expression in transgenic wheat

The contiguous crCas9 and sgRNA blocks cassette in the recombinant, modified pCambia 1302 vector was amplified using primers including the Not1 and Asc1restriction sites (Additional file 11) and cloned into the Gateway entry vector pENTR/D-TOPO (Thermo Fisher Scientific). Two Gateway reactions using the LR Clonase Plus (Thermo Fisher Scientific) were required to recombine the Cas9 - sgRNA blocks cassette as well as a wheat ubiquitin promoter into the binary plant transformation destination vector pVB29 (Additional file 9); pVB29 is a modified pPZP200 vector (SnapGene, Chicago) containing the phosphinothricin acetyltransferase (PAT) gene for Basta resistance, controlled by an additional wheat ubiquitin promoter. This modified vector pVB29 was transformed into Stellar *E. coli* competent cells (Clontech) and primers containing the sgRNA specific sequences were used for sequencing to confirm identity and integrity of the construct. Plant transformation was performed into Fielder by particle bombardment using an established protocol based on [40]. Green shoots obtained from selection on phosphinothricin (L-PPT) at 2.5 mg/L were rooted on 5 mg/L L-PPT and the transgenic plants were transferred to soil and grown in cabinets. The T1 progeny from four plants expressing Cas9 and sgRNA were grown in controlled-environment cabinets with 16 h light at 20 °C and 8 h dark at 16 °C. Leaf tissues (about 8 cm leaf pieces) were collected from 2-week old seedlings and DNA extracted using the DNeasy 96 Plant Kit (Qiagen) according to manufacturer instructions. DNA concentrations were determined fluorometrically using Quant-iT dsDNA Assay kit (Invitrogen/Thermo Fisher Scientific) and FLUOstar Omega microplate reader (BMG LABTECH/Mandel Scientific Co, Guelph, Canada).

### Genotyping of transgenic wheat to identify editing events in *TaNFXL1* homoeologous genes

A rapid, economic genotyping method was designed to identify editing events in the three wheat subgenomes, adapted from Schuelke [20] (Fig. 2). For a first PCR reaction, primers specific to each of the six *TaNFXL1* homoeologous genes were designed from the sequences flanking the target sites for both sgRNA using the wheat genomic sequence RefSeq v1.0 [31] (Additional file 11). The primers were designed with a SNP at the 3′ end of each forward and reverse primer, and whenever possible additional SNP within the primer sequence, allowing homoeolog-specific amplifications. Care was also taken to position the primers in such a manner that different fragment sizes would be amplified. The universal primer sequence CAGTCGGGCGTCATCACAC was added at the 5′ end of each forward primer sequence. The primers were used for a first, touchdown PCR using Q5 Reaction Buffer (New England BioLab Inc.), 0.2 mM dNTPs, 0.5 µM each of homoeolog-specific forward and reverse primers, 0.2 U of Q5 High-Fidelity DNA polymerase (New England BioLab Inc) and 240 ng genomic DNA from individual T1 progeny in a 10 µL final volume, with the following amplification conditions: denaturation at 98 °C for 3 min followed by 10 cycles of 98 °C for 10 s, 68 °C (with gradual 1 °C per cycle temperature reduction until it reached 58 °C) for 30 s, 72 °C for 30 s; followed by 30 cycles of 98 °C for 10 s, 58 °C for 30 s, 72 °C for 30 s, and a final extension step at 72 °C for 5 min. PCR products were cleaned up by mixing 3 μL of a PCR reaction product with 1 μL of ExoSAP-IT PCR Product Cleanup Reagent (Thermo Fisher Scientific) and proceeding as per manufacturer’s instructions. FAM-, NED-, VIC-fluorescence-labelled versions of the universal primer were synthetized by Thermo Fisher Scientific. The amplified fragments from the subgenome A, B and D homoeologs of the gene X group (or of the gene Y group) were respectively labeled with the FAM, NED and VIC fluorescent dyes in a second PCR reaction using 0.5 µM of a labeled universal primer together with the appropriate homoeolog-specific reverse primer and 1 µL of first reaction PCR product. Similar touchdown PCR conditions as for the first round were used, except that only 25 cycles of amplification were performed once the annealing temperature reached 58 °C. The PCR products labeled with FAM-, NED-, and VIC- for each gene group were mixed together and separated by capillary electrophoresis on an IBI 3500 Genetic Analyzer with 8-Capillary Array. GeneScan 600 LIZ (20-600 nucleotides, Applied BioSystems) was used as internal size standard. Patterns of amplification profiles were analysed by GeneMapper v5 (Thermo Fisher Scientific).

## Supplementary information

**Additional file 1.** Homoeologous genes from wheat genomic sequence RefSeq v1.0 [9] best matching with *TaABCC6*, *TansLTP9.4* and *TaNFXL1*. Sequences from Fielder for each of the homoeolog that match to each sgRNA are provided with the number (right column) and identity (in bold) of mismatched bases, when occurring. For *TaABCC6* and *TaNFXL1*, gene X group includes the homoeologs with the best match (97–100% identity) to the NCBI accession used to design the probe sets on the microarray, while gene Y group corresponds to very closely related homoeologs (92-95% identity). * indicates the genes that were not or very poorly (<15 reads) amplified in this study with the primers used.

**Additional file 2.** *In vitro* testing of six sgRNA for *TaABCC6* (A), *TansLTP9.4* (B) and *TaNFXL1* (C). 1 kb Plus DNA Ladder (M) was used in all three gel electrophoresis and digested fragments are marked with red arrowheads. Each panel shows the uncleaved DNA template (1), digestions using Cas9 guided by sgRNA-1 (2) and sgRNA-2 (3).

**Additional file 3.** Schematic representation of the region of modified pCambia 1302 vector containing either crCas9 [6] or pcoCas9 [19] and the elements of the inserted gBlock pair fragment. TaU6 is from [32]. pCambia 1302 itself, not shown, is located between the right (RB) and left (LB) border elements.

**Additional file 4.** Wheat protoplasts transformed with pMDC32-ZsGreen and visualised in dark field, revealing the green fluoresce (A), and in bright field (B). The observation and the counting were performed 48 h after transformation.

**Additional file 5.** Protoplast transformation experiments used for the preparation of PCR amplicons for HTS.

**Additional file 6.** Structure of the PCR-amplified regions for *TaABCC6*, *TansLTP9.4* and *TaNFXL1* genes. Introns are indicated by horizontal lines; rectangular boxes represent exons. Positions of sgRNA are marked by vertical yellow lines.

**Additional file 7.** Complete list of editing observed in the protoplast system. Editing include modifications identified by Breakpoint analysis on target regions in samples (A) ABCC6-1 to -5, (B) nsLTP9.4-1 to -4, (C) NFXL1-1 to -4 and (D) pcoNFXL1-1 to -5 at either sgRNA1 or sgRNA2 site; (E) deletions between sgRNA pairs identified by InDel analysis for ABCC6-1 to -5, nsLTP9.4-1 to -4 and pcoNFXL1-4 and (F) replacements identified by Structural Variant analysis for NFXL1-1 to -4 and pcoNFXL1-1 to -4. Only samples where editing of one type or another were found are mentioned. In (E), the “Deleted sequence” refers to the deleted sequences between two sgRNA sites. Frequency= (Reads with modification)/ (Mapped reads + Reads with modification).

**Additional file 8.** Estimated editing efficiency in the protoplast system at the individual target sites for sgRNA-1 and sgRNA-2.

**Additional file 9.** Vector used for wheat transformation. (A) Cloning strategy using the Gateway recombination system to introduce two fragments into the vector pVB29. (B) Final vector used for wheat microprojectile bombardment.

**Additional file 10.** Size (bp) of genotyping amplicons observed for each of the six wheat homoeologs of *TaNFXL1* in the T1 progeny of plants transformed with a construct expressing Cas9 and two sgRNA. To be conservative with the naming of the edited alleles, amplicons varying by less than 5 bp in size were given the same name. See Table 4 for an explication of the genotype code. N/A in amplicon size columns and ? in the genotype code indicate results not available. Genotype for Fielder, the parent line for the transgenic plants, is highlighted in grey.

**Additional file 11.** Sequence of the primers used in this study.

**Additional file 12.** gBlock components. (A) Schematic representation of components in a gBlock. Overlap: sequences used for Gibson assembly or cloning; U6 promoter: wheat U6 promoter [32] for transcription of sgRNA and sgRNA scaffold. (B) Full sequences for two gBlocks (gBlocks 1 and 2; 546 bp for one block). In italic: 40 bp overlap with the pCambia vector; underlined: wheat U6 promoter; 20 Ns in blue: position of sgRNA sequence; in green: sgRNA scaffold and terminator; in red: 40 bp overlapping sequence between the two gBlocks for Gibson assembly [34].

**Additional file 13.** Map of the pMDC32-ZsGreen vector. 2x…moter: 2x 35S promoter; ZsGreen: green fluorescent protein from [37]; n…r: NOS terminator; Hygromycin: hygromycin resistance gene; Kan: kanamycin resistance gene.

## Data Availability

The datasets supporting the conclusions of this article are included within the article (and its supplementary information files).

## Abbreviations

Bp: base pair
CRISPR: clustered regularly interspaced short palindromic repeats
HTS: high throughput sequencing
Kbp: kilobase pair
sgRNA: single guide RNA
SNP: single nucleotide polymorphism
T1 progeny: first generation from transgenic plants
