# Correction to: An optimised CRISPR/Cas9 protocol to create targeted mutations in homoeologous genes and an efficient genotyping protocol to identify edited events in wheat

**DOI:** 10.1186/s13007-019-0539-0

**Published:** 2019-12-30

**Authors:** Xiucheng Cui, Margaret Balcerzak, Johann Schernthaner, Vivijan Babic, Raju Datla, Elizabeth K. Brauer, Natalie Labbé, Rajagopal Subramaniam, Thérèse Ouellet

**Affiliations:** 1 Ottawa Research and Development Centre, 960 Carling Avenue, Ottawa, ON K1A 0C6 Canada; 2 Department of Biology, University of Ottawa, 75 Laurier Ave E, Ottawa, ON K1N 6N5 Canada; 3 Aquatic and Crop Resource Development, National Research Council Canada, 110 Gymnasium Place, Saskatoon, SK S7N 0W9 Canada

## Correction to: Plant Methods (2019) 15:119 10.1186/s13007-019-0500-2

In the original publication [1], the copyright line was incorrectly published as “© The Author(s) 2019”. The corrected copyright line should read as “© Her Majesty the Queen in Right of Canada as represented by the Minister of Agriculture and Agri-Food Canada, 2019”. The original article has been corrected.
