# Supplementary material for: An optimised CRISPR/Cas9 protocol to create targeted mutations in homoeologous genes and an efficient genotyping protocol to identify edited events in wheat
Source: Plant Methods. 2019 Oct 24;15:119. doi: 10.1186/s13007-019-0500-2 (PMC6814032; doi:10.1186/s13007-019-0500-2)
Supplement: Supplementary file 1 — Additional file 1. Homoeologous genes from wheat genomic sequence RefSeq v1.0 [9] best matching with TaABCC6, TansLTP9.4 and TaNFXL1. Sequences from Fielder for each of the homoeolog that match to each sgRNA are provided with the number (right column) and identity (in bold) of mismatched bases, when occurring. For TaABCC6 and TaNFXL1, gene X group includes the homoeologs with the best match (97–100% identity) to the NCBI accession used to design the probe sets on the microarray, while gene Y group corresponds to very closely related homoeologs (92-95% identity). * indicates the genes that were not or very poorly (<15 reads) amplified in this study with the primers used. [file 13007_2019_500_MOESM1_ESM.docx]

**TaABCC6**  Mismatch

Group sgRNA-1 CACGCCGTCGAGATTACTGG

Gene X TraesCS2A01G451300 CACGCCGTCGAGATTACTGG 0

TraesCS2B01G472800 CACGCCGTC**A**AGATTACTGG 1

TraesCS2D01G451100 CACGCCGTCGAGATTACTGG 0

Gene Y TraesCS2A01G451500 CACGCC**A**TC**A**AGATTACTGG 2

TraesCS2B01G473000* CACGCCGTCGAGATTACTGG 0

TraesCS2D01G451300 CACGCCGTC**A**AGATTACTGG 1

Group sgRNA-2 AGTACTCACGGAGATCCAAG

Gene X TraesCS2A01G451300 AGTACTCACGGAGATCCAAG 0

TraesCS2B01G472800 AGTACTC**G**CGGAGATCCAAG 1

TraesCS2D01G451100 AGTACTCACGGAGATCCAAG 0

Gene Y TraesCS2A01G451500 AGTACTCAC**A**GAGATCCAAG 1

TraesCS2B01G473000* AGTACTC**G**CGGAGATCCAAG 1

TraesCS2D01G451300 AGTACTCACGGAGATCCAAG 0

**TaNFXL1**

Group sgRNA-1 TGACTGGCACAACGCAAGGT

Gene X TraesCS7A01G518800 TGACTGGCACAACGCAAGGT 0

TraesCS7B01G434700 TGACTGGCACAACGCAAGGT 0

TraesCS7D01G508800* TGACTGGCACAACGCAAGGT 0

Gene Y TraesCS7A01G733000LC* TGACTGGCACAACGCAAGGT 0 TraesCS7B01G432300* TGACTGGCACAACGCAAGGT 0

TraesCS7D01G688900LC T**T**ACTG**A**CACAAC**A**CAAGGT 3

Group sgRNA-2 GATGGAGTTGGTGTGCCGCA

Gene X TraesCS7A01G518800 GATGGAGTTGGTGTGCCGCA 0

TraesCS7B01G434700 GATGGAGTTGGTGTGCCGCA 0

TraesCS7D01G508800* GATGGAGTTGGTGTGCCGCA 0

Gene Y TraesCS7A01G733000LC* GATGGAGTTGGTGTGCCGCA 0

TraesCS7B01G432300* GATGGAGTTGGTGTGCCGCA 0

TraesCS7D01G688900LC GATGGAGTTGGTGTGCCGCA 0

**nsLTP9.4***

Group sgRNA-1 GCCGTGCGTGGCGTACGTGA

Gene X TraesCS5A01G147000 GCCGTGCGTGGCGTACGTGA 0

TraesCS5B01G145900* GCCGTGCGTGGCGTACGTGA 0

TraesCS5D01G145300* GCCGTGCGTG**T**CGTACGTGA 1

sgRNA-2 AGTGCTGCTCCGGCGTGCAG

Gene X TraesCS5A01G147000 AGTGCTGCTCCGGCGTGCAG 0

TraesCS5B01G145900* AGTGCTGCTCCGGCGTGCAG 0

TraesCS5D01G145300* AGTGCTGCTCCGGCGTGCAG 0

* Locus-specific fragment was not or very poorly (<15 reads) amplified in this study with the primers used.
