# Supplementary figures and images for: An optimised CRISPR/Cas9 protocol to create targeted mutations in homoeologous genes and an efficient genotyping protocol to identify edited events in wheat
Source: Plant Methods. 2019 Oct 24;15:119. doi: 10.1186/s13007-019-0500-2 (PMC6814032; doi:10.1186/s13007-019-0500-2)

## Slide 1
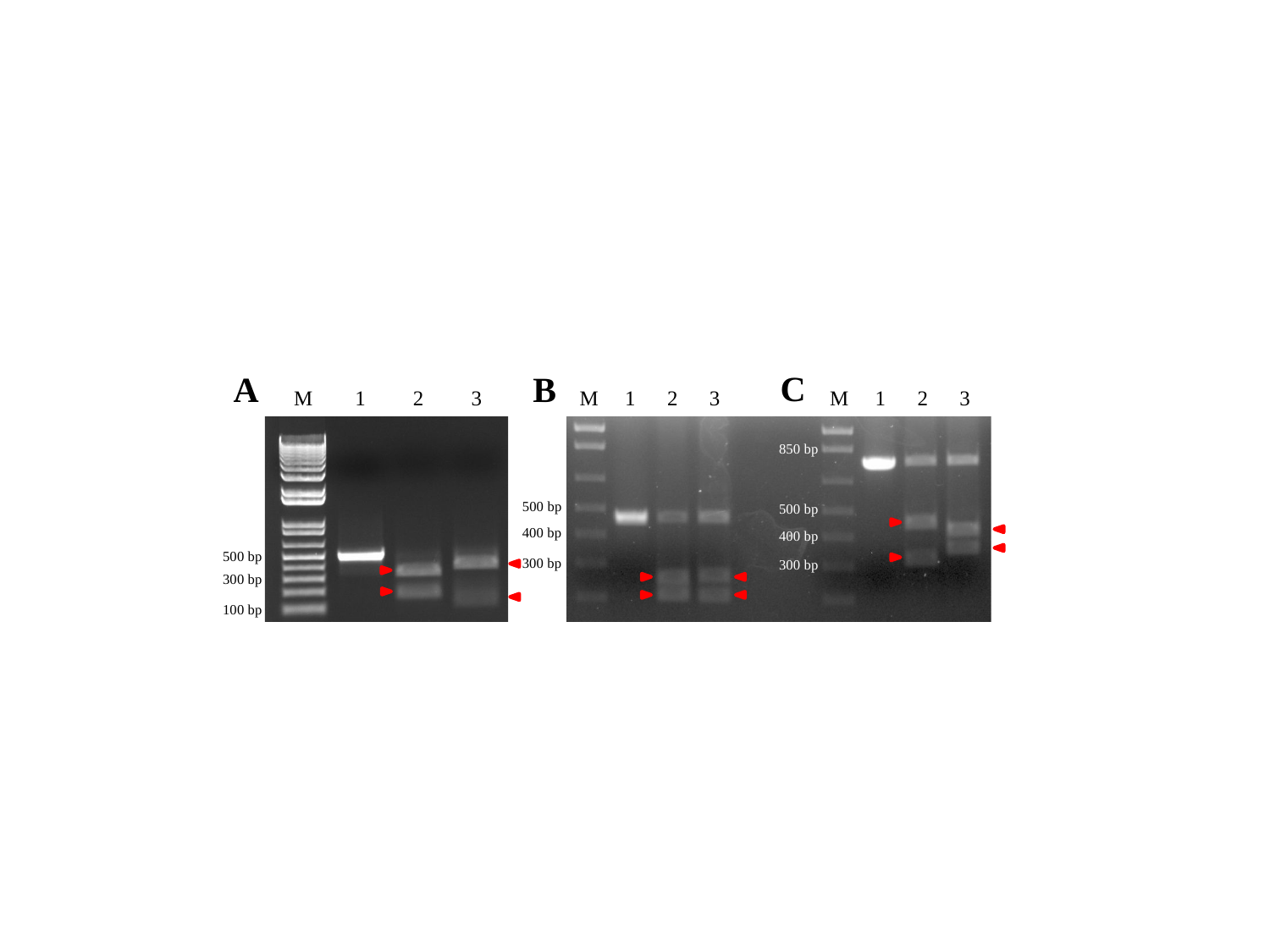

C
A
B
M 1 2 3
M 1 2 3
850 bp
500 bp
400 bp
300 bp
500 bp
400 bp
300 bp
M 1 2 3
500 bp
300 bp
100 bp

Supplement: Supplementary file 2 — Additional file 2. In vitro testing of six sgRNA for TaABCC6 (A), TansLTP9.4 (B) and TaNFXL1 (C). 1 kb Plus DNA Ladder (M) was used in all three gel electrophoresis and digested fragments are marked with red arrowheads. Each panel shows the uncleaved DNA template (1), digestions using Cas9 guided by sgRNA-1 (2) and sgRNA-2 (3). [file 13007_2019_500_MOESM2_ESM.pptx]

## Slide 1
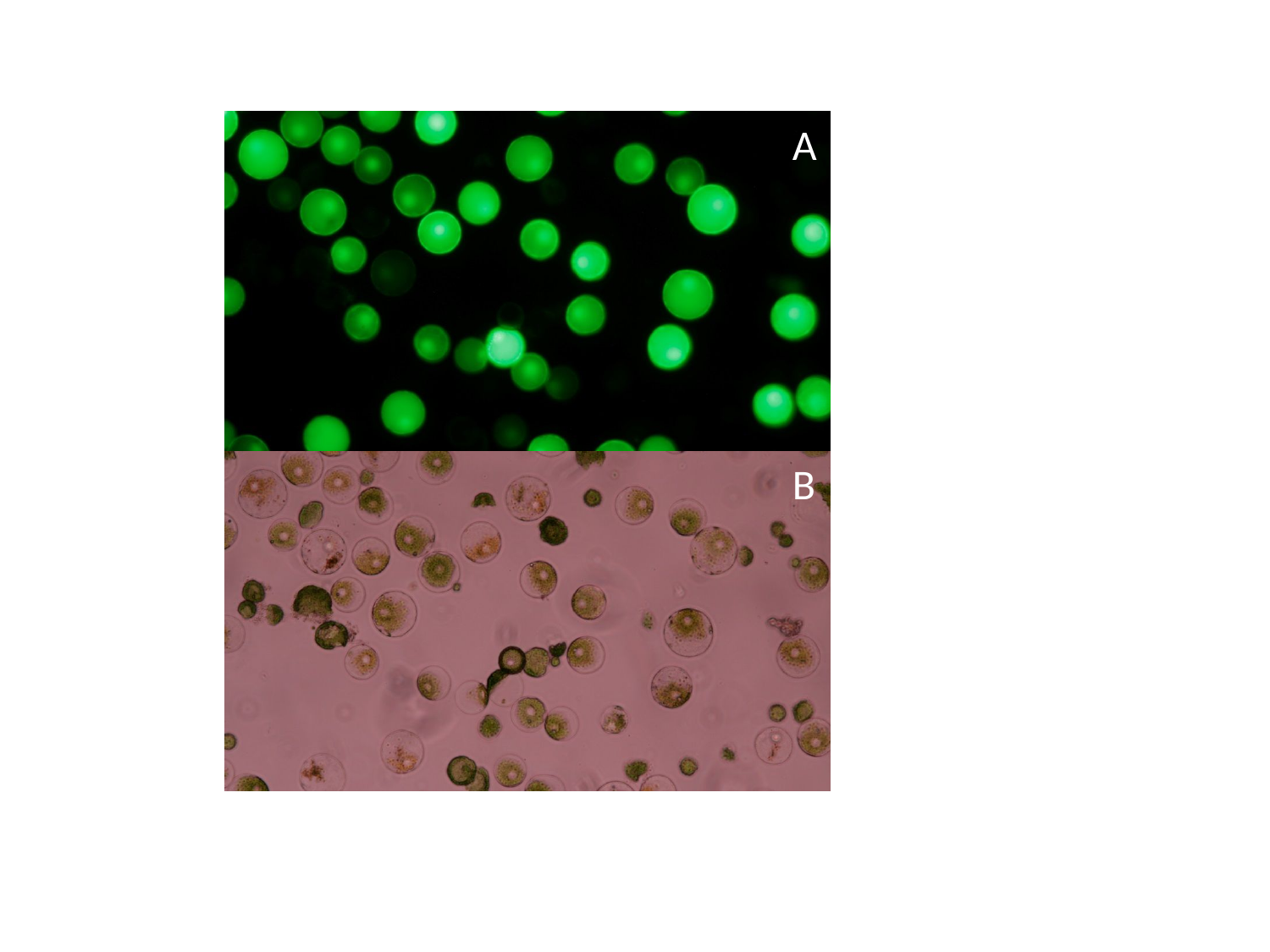

A
B

Supplement: Supplementary file 4 — Additional file 4. Wheat protoplasts transformed with pMDC32-ZsGreen and visualised in dark field, revealing the green fluoresce (A), and in bright field (B). The observation and the counting were performed 48 h after transformation. [file 13007_2019_500_MOESM4_ESM.pptx]

## Slide 1
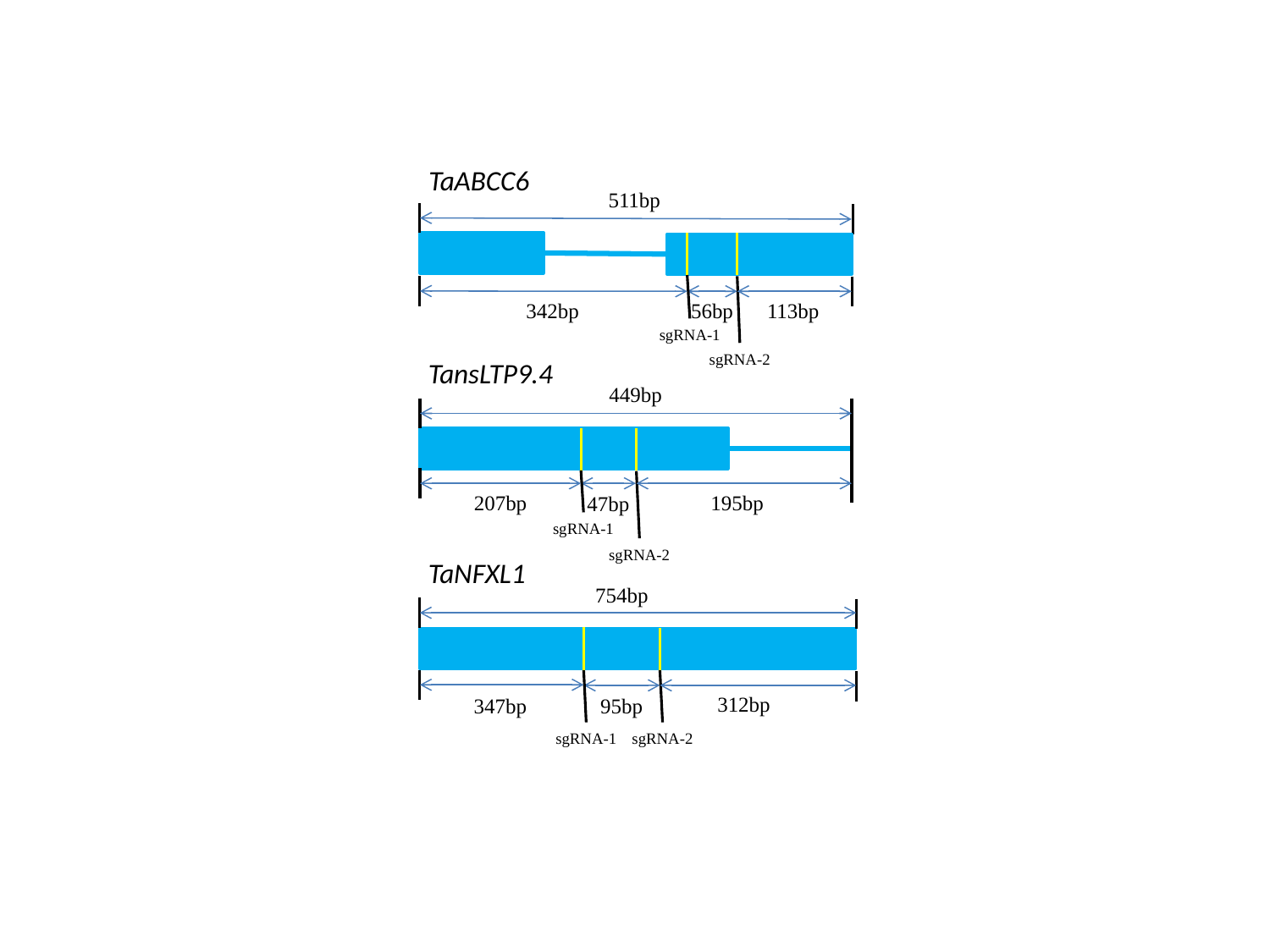

TaABCC6
511bp
56bp
342bp
113bp
sgRNA-1
sgRNA-2
449bp
195bp
207bp
47bp
sgRNA-1
sgRNA-2
754bp
312bp
347bp
95bp
sgRNA-1
sgRNA-2
TansLTP9.4
TaNFXL1

Supplement: Supplementary file 6 — Additional file 6. Structure of the PCR-amplified regions for TaABCC6, TansLTP9.4 and TaNFXL1 genes. Introns are indicated by horizontal lines; rectangular boxes represent exons. Positions of sgRNA are marked by vertical yellow lines. [file 13007_2019_500_MOESM6_ESM.pptx]

## Slide 1
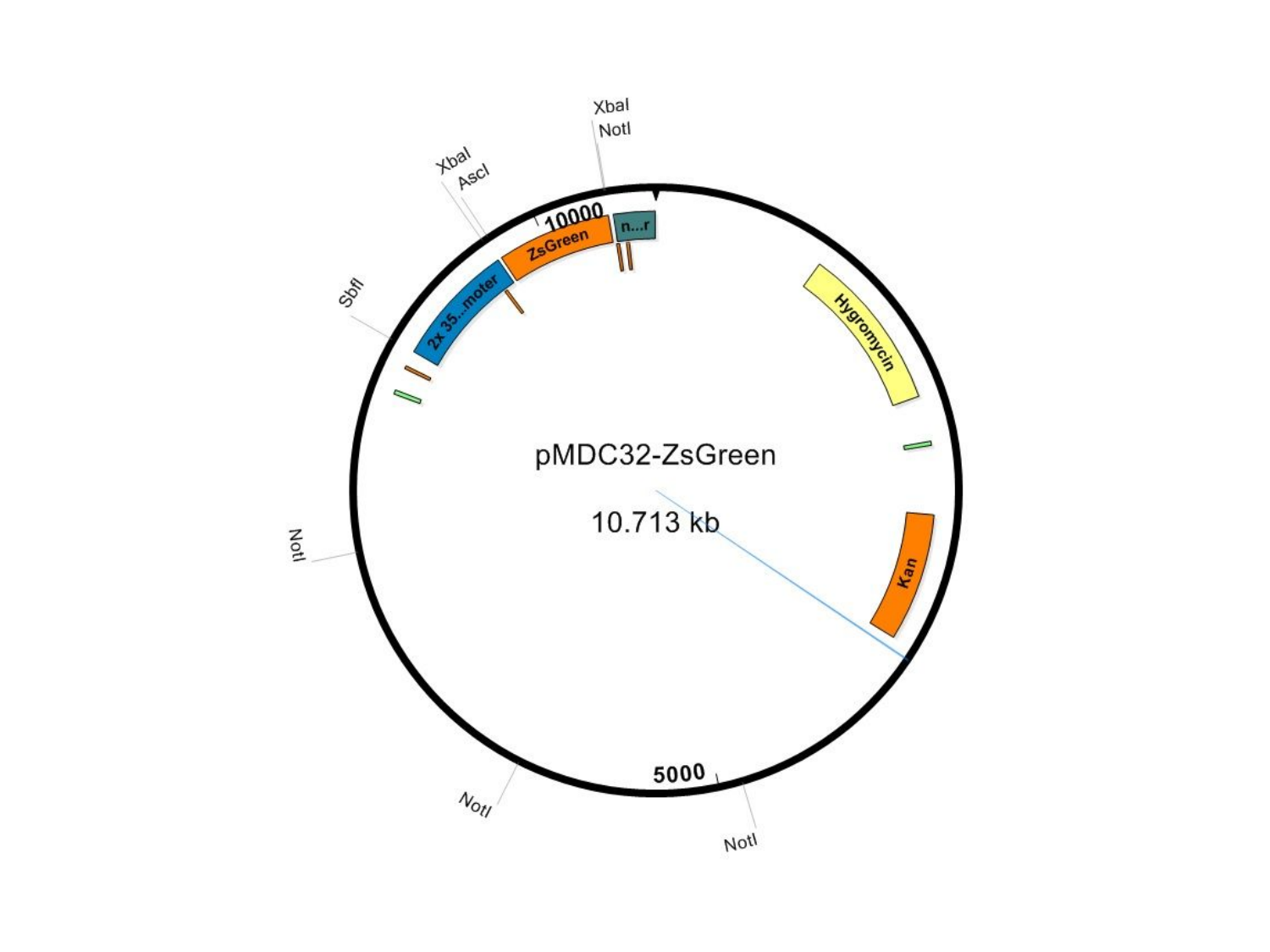

Supplement: Supplementary file 13 — Additional file 13. Map of the pMDC32-ZsGreen vector. 2x…moter: 2x 35S promoter; ZsGreen: green fluorescent protein from [37]; n…r: NOS terminator; Hygromycin: hygromycin resistance gene; Kan: kanamycin resistance gene. [file 13007_2019_500_MOESM13_ESM.pptx]
