# Supplementary material for: An optimised CRISPR/Cas9 protocol to create targeted mutations in homoeologous genes and an efficient genotyping protocol to identify edited events in wheat
Source: Plant Methods. 2019 Oct 24;15:119. doi: 10.1186/s13007-019-0500-2 (PMC6814032; doi:10.1186/s13007-019-0500-2)
Supplement: Supplementary file 3 — Additional file 3. Schematic representation of the region of modified pCambia 1302 vector containing either crCas9 [6] or pcoCas9 [19] and the elements of the inserted gBlock pair fragment. TaU6 is from [32]. pCambia 1302 itself, not shown, is located between the right (RB) and left (LB) border elements. [file 13007_2019_500_MOESM3_ESM.pptx]

## Slide 1
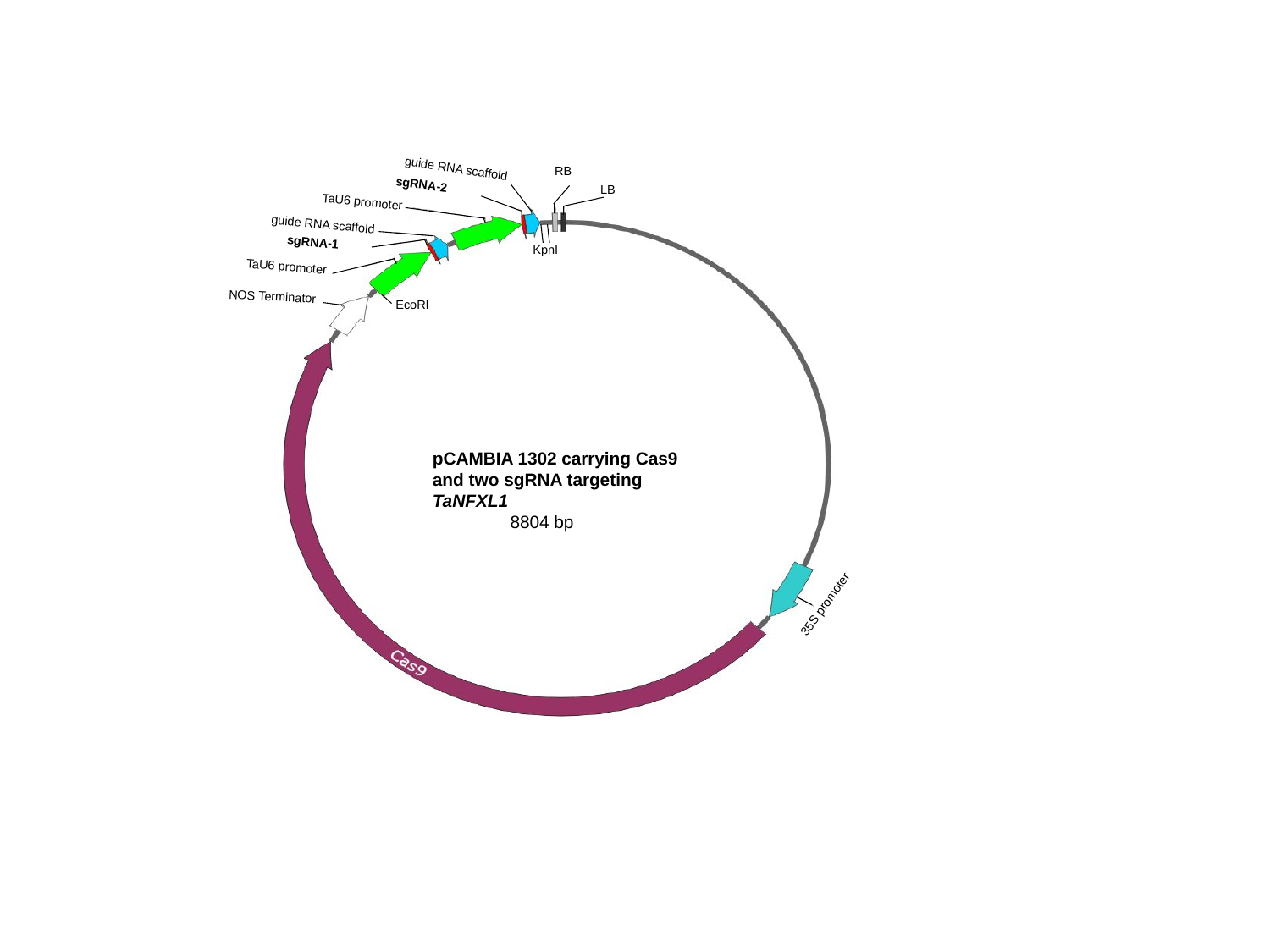

guide RNA scaffold
RB
sgRNA-2
LB
TaU6 promoter
guide RNA scaffold
sgRNA-1
TaU6 promoter
NOS Terminator
pCAMBIA 1302 carrying Cas9 and two sgRNA targeting TaNFXL1
 8804 bp
35S promoter
KpnI
EcoRI
