# Supplementary material for: An optimised CRISPR/Cas9 protocol to create targeted mutations in homoeologous genes and an efficient genotyping protocol to identify edited events in wheat
Source: Plant Methods. 2019 Oct 24;15:119. doi: 10.1186/s13007-019-0500-2 (PMC6814032; doi:10.1186/s13007-019-0500-2)
Supplement: Supplementary file 5 — Additional file 5. Protoplast transformation experiments used for the preparation of PCR amplicons for HTS. [file 13007_2019_500_MOESM5_ESM.docx]

**Additional file 5**. Protoplast transformation experiments used for the preparation of PCR amplicons for HTS.

| Experiment | DNA sample name^a^ | |
| --- | --- | --- |
| 1 | nsLTP9.4-1,-2 | NFXL1-1,-2 |
| 2 | nsLTP9.4-3,-4 | NFXL1-3,-4 |
| 3 | nsLTP9.4-5 | NFXL1-5 |
| 4 | ABCC6-1,-2 | pcoNFXL1-1,-2 |
| 5 | ABCC6-3,-4 | pcoNFXL1-3,-4 |
| 6 | ABCC6-5, control | pcoNFXL1-5, control |

^a^Transformations were done with the vectors expressing crCas9 except for the samples with a name starting with pco, which were transformed with the vector containing pcoCas9. For each sample, two individual transformations were pooled together for DNA extraction; samples from the same experiment were from the same batch of protoplasts. Control, non-transformed Fielder protoplasts
