# Supplementary material for: An optimised CRISPR/Cas9 protocol to create targeted mutations in homoeologous genes and an efficient genotyping protocol to identify edited events in wheat
Source: Plant Methods. 2019 Oct 24;15:119. doi: 10.1186/s13007-019-0500-2 (PMC6814032; doi:10.1186/s13007-019-0500-2)
Supplement: Supplementary file 7 — Additional file 7. Complete list of editing observed in the protoplast system. Editing include modifications identified by Breakpoint analysis on target regions in samples (A) ABCC6-1 to -5, (B) nsLTP9.4-1 to -4, (C) NFXL1-1 to -4 and (D) pcoNFXL1-1 to -5 at either sgRNA1 or sgRNA2 site; (E) deletions between sgRNA pairs identified by InDel analysis for ABCC6-1 to -5, nsLTP9.4-1 to -4 and pcoNFXL1-4 and (F) replacements identified by Structural Variant analysis for NFXL1-1 to -4 and pcoNFXL1-1 to -4. Only samples where editing of one type or another were found are mentioned. In (E), the “Deleted sequence” refers to the deleted sequences between two sgRNA sites. Frequency= (Reads with modification)/ (Mapped reads + Reads with modification). [file 13007_2019_500_MOESM7_ESM.docx]

**Additional file 7.** Complete list of editing observed in the protoplast system. Editing include modifications identified by Breakpoint analysis on target regions in samples (A) ABCC6-1 to -5 , (B) nsLTP9.4-1 to -4, (C) NFXL1-1 to -4 and (D) pcoNFXL1-1 to -5 at either sgRNA1 or sgRNA2 site; (E) deletions between the sgRNA pairs identified by InDel analysis for samples ABCC6-1 to -5, nsLTP9.4-1 to -4 and pcoNFXL1-4 and (F) replacements identified by Structural Variant analysis for samples NFXL1-1 to -4 and pcoNFXL1-1 to -4. Only samples where editing of one type or another were found are mentioned. In (E), the “Deleted sequence” refers to the deleted sequences between two sgRNA sites. Frequency= (Reads with modification)/ (Mapped reads + Reads with modification).

**A**

| Sample | p-value | Modified sequence (Deleted/ Inserted) | Length (bp) | Editing type | Mapped reads | Reads with modification | Frequency |
| --- | --- | --- | --- | --- | --- | --- | --- |
| ABCC6-1 | 1.55E-07 | AGGGGTTCTAAAGCACCACAAACAATTGAACGGCGGCGAAACCGGCATGAAGCCAACTAGGCGCACCGCAGAACCCAGC | 79 | Deletion | 3686 | 8 | 0.002166 |
| ABCC6-1 | 0 | AAGGGGTTCTAAAGCACCACAAACAATTGAACGGCGGCGAAACCGGCATGAAGCCAACTAGGCGCACCGCAGAACCCAGCA | 81 | Deletion | 3696 | 24 | 0.006452 |
| ABCC6-1 | 0 | AGCTGCTGAAGCGCTAAGATCTGTACTCGTTTGTGGTGAGCAGGAAGGATTATAGAATACGACACGCCGTCGAGATTA | 78 | Deletion | 3965 | 21 | 0.005268 |
| ABCC6-1 | 1.04E-05 | AGCTGCTGAAGCGCTAAGATCTGTACTCGTTTGTGGTGAGCAGGAAGGATTATAGAATACGACACGCCGTCGAGATT | 77 | Deletion | 3960 | 7 | 0.001765 |
| ABCC6-2 | 2.07E-06 | TGAAGCGCTAAGATCTGTACTCGTTTGTGGTGAGCAGGAAGGATTATAGAATACGACACGCCGGCGAGATTA | 72 | Deletion | 6267 | 8 | 0.001275 |
| ABCC6-3 | 9.45E-14 | AAGGGGTTCTAAAGCACCACAAACAATTGAACGGCGGCGAAACCGGCATGAAGCCAACTAGGCGCACCGCAGAACCCAGCA | 81 | Deletion | 4494 | 14 | 0.003106 |
| ABCC6-3 | 3.13E-12 | CCTAGCTGCTGAAGCGCTAAGATCTGTACTCGTTTGTGGTGAGCAGGAAGGATTATAGAATACGACACGCCGTCGAGATTA | 81 | Deletion | 4661 | 14 | 0.002995 |
| ABCC6-4 | 0 | AAGGGGTTCTAAAGCACCACAAACAATTGAACGGCGGCGAAACCGGCATGAAGCCAACTAGGCGCACCGCAGAACCCAGCA | 81 | Deletion | 4794 | 95 | 0.019431 |
| ABCC6-4 | 0 | GCCACAGCACCGACGAACCCAAAATATTTATCCACATTTTAGGACCTAGCTGCTGCAGTGCTAAGATCCGTACTCGTTTGTGCTGAGCAGGAAGAATTATAGAATACGACACGCCGTCGAGATTA | 125 | Deletion | 5052 | 56 | 0.010963 |
| ABCC6-4 | 2.41E-10 | CTACATTTTAGGACCTAGCTGCTGAAGCGCTAAGATCTGTACTCGTTTGTGGTGAGCAGGAAGGATTATA | 70 | Deletion | 5046 | 14 | 0.002767 |
| ABCC6-5 | 1.44E-15 | AGCCAGGTCATCGAAGAACCCAAAATATTTATCCACATTTTAGGACCTAGCTGCTGCAGTGCTAAGATCCGTACTCGTTTGTGCTGAGCAGGAAGAATTATAGAATACGACACGCCGTCGAGATTA | 126 | Deletion | 6131 | 19 | 0.003089 |

B

| Sample | p-value | Modified sequence (Deleted/ Inserted) | Length (bp) | Editing type | Mapped reads | Reads with modification | Frequency |
| --- | --- | --- | --- | --- | --- | --- | --- |
| nsLTP9.4-1 | 0 | GGTGGACTCCAAGCTCGCGCCGTGCGTGGCGTAC | 34 | Deletion | 16519 | 55 | 0.003318 |
| nsLTP9.4-1 | 2.15E-06 | GGCGTACG | 8 | Deletion | 12939 | 4 | 0.000309 |
| nsLTP9.4-1 | 0 | CAGGTGGACTCCAAGCTCGCGCCGTGCGTGGCGTAC | 36 | Deletion | 16614 | 25 | 0.001502 |
| nsLTP9.4-2 | 0 | GGTGGACTCCAAGCTCGCGCCGTGCGTGGCGTAC | 34 | Deletion | 19650 | 28 | 0.001423 |
| nsLTP9.4-2 | 0 | CAGGTGGACTCCAAGCTCGCGCCGTGCGTGGCGTAC | 36 | Deletion | 19735 | 14 | 0.000709 |
| nsLTP9.4-3 | 3.49E-14 | GGCGTACG | 8 | Deletion | 17370 | 13 | 0.000748 |
| nsLTP9.4-3 | 5.12E-09 | CGTGGCGTACG | 11 | Deletion | 18210 | 9 | 0.000494 |
| nsLTP9.4-3 | 3E-07 | GCGGGCAGGTGGACTCCAAGCTCGCGCCGT | 30 | Deletion | 22021 | 8 | 0.000363 |
| nsLTP9.4-3 | 0 | CAGGTGGACTCCAAGCTCGCGCCGTGCGTGGCGTAC | 36 | Deletion | 22577 | 299 | 0.01307 |
| nsLTP9.4-3 | 0 | CAGGTGGACTCCAAGCTCGCGCCGTGCGTGGCGTAC | 36 | Deletion | 22720 | 60 | 0.002634 |
| nsLTP9.4-3 | 6.65E-14 | CAGGTGGACTCCAAGCTCGCGCCGTGCGTGGCGTACG | 37 | Deletion | 22846 | 14 | 0.000612 |
| nsLTP9.4-4 | 2.48E-10 | CGCCGTGCGTGGCGTAC | 17 | Deletion | 12211 | 9 | 0.000736 |
| nsLTP9.4-4 | 6E-15 | GTGGACTCCAAGCTCGCGCCGTGCGTGGCGTAC | 33 | Deletion | 14391 | 13 | 0.000903 |
| nsLTP9.4-4 | 0 | GGTGGACTCCAAGCTCGCGCCGTGCGTGGCGTAC | 34 | Deletion | 14457 | 235 | 0.015995 |
| nsLTP9.4-4 | 0 | CAGGTGGACTCCAAGCTCGCGCCGTGCGTGGCGTAC | 36 | Deletion | 14538 | 39 | 0.002675 |

**C**

| Sample | p-value | Modified sequence (Deleted/ Inserted) | Length (bp) | Editing type | Mapped reads | Reads with modification | Frequency |
| --- | --- | --- | --- | --- | --- | --- | --- |
| NFXL1-1 | 0 | CGCAAGGCTGTGGTGGCGGAATAGATGTCCTGCCACAGGCACAAGTGAGATCGTGGAATATGGTCTCACGGCAAGGGTCGCAATGACCGCTGTGGC | 96 | Deletion | 9702 | 138 | 0.014024 |
| NFXL1-1 | 0 | CAAGGCTGTGGTGGCGGAATAGATGTCCTGCCACAGGCACAAGTGAGATCGTGGAATATGGTCTCACGGCAAGGGTCGCAATGACCGCTGTGGC | 94 | Deletion | 9706 | 124 | 0.012614 |
| NFXL1-1 | 0 | GCATCTGATATCCTTTGAACCACAGGGGATGTTCCTCAACATCACATGCCCCCCAACACATTCTCTTGTGACTGGCACAACGCAA | 85 | Deletion | 11242 | 91 | 0.00803 |
| NFXL1-1 | 0 | CATCTGATATCCTTTGAACCACAAGGGATGTTCCTCAACATCACATGCCCCCCAACACATTCTCTTGTGACTGGCACAACGCAA | 84 | Deletion | 11261 | 111 | 0.009761 |
| NFXL1-2 | 3.32E-11 | GCAAGGCTGTGGTGGTGGAATGGATGTCCTGCCACAGGCACAAGTGAGATCATGGAATATGGTCTCACGGCAAGGGTCGCAATGACCGCTGTGG | 94 | Deletion | 18600 | 27 | 0.00145 |
| NFXL1-2 | 0 | CGCAAGGCTGCGGTGGCGGAATAGATGACCTGCCACACGCACAAGAGAGATCATTGAATATGGTCTCACGGCAAGGGTCGCAGTGACCGCTGTGGCAGAGATGCTGGCATCCACCTGTCTCTTATA | 126 | Deletion | 18537 | 625 | 0.032617 |
| NFXL1-2 | 0 | CAAGGCTGTGGTGGCGGAATAGATGTCCTGCCACAGGCACAAGTGAGATCGTGGAATATGGTCTCACGGCAAGGGTCGCAATGACCGCTGTGGC | 94 | Deletion | 18556 | 619 | 0.032282 |
| NFXL1-2 | 0 | TGCATCTGATATCCTTTGAACCACAAGGGACGTTCCTCAACATCACATGCCCCCCAACACATTCTCTTGTGACTGGCACAACGCAA | 86 | Deletion | 22890 | 363 | 0.015611 |
| NFXL1-2 | 0 | TATAAGAGGACAGAGCTTTCCACAGGGCTGGTTGCATCTGATATCCTTTGAACCACAGGGGATGTTCCTTAGCATCACATGTCCTCCAACGCACTCTCTCATGACTGGCACAACGCAA | 118 | Deletion | 22903 | 528 | 0.022534 |
| NFXL1-2 | 0 | TGCATCTGATATCCTTTGAACCACAAGGGATGTTCCTCAACATCACATGCCCCCCAACACATTCTCTTGTGACTGGC | 77 | Deletion | 22414 | 148 | 0.00656 |
| NFXL1-3 | 1.48E-12 | GGCAAGGCTGTGGTGGCGGAATAGATGTCCTGCCACAGGCACAAGTGAGATCGTGGAATATGGTCTCACGGCAAGGGTCGCAATGACCGCTGTGGC | 96 | Deletion | 31229 | 62 | 0.001981 |
| NFXL1-3 | 0 | GCAAGGCTGTGGTGGCGGAATAGATGTCCTGCCACAGGCACAAGTGAGATCGTGGAATATGGTCTCACGGCAAGGGTCGCAATGACCGCTGTGGC | 95 | Deletion | 31308 | 545 | 0.01711 |
| NFXL1-3 | 0 | CGCAAGGCTGTGGTGGCGGAATAGATGTCCTGCCACAGGCACAAGTGAGATCGTGGAATATGGTCTCACGGCAAGGGTCGCAATGACCGCTGTGGC | 96 | Deletion | 31263 | 145 | 0.004617 |
| NFXL1-3 | 0 | CGCAAGGCTGTGGTGGCGGAATAGATGTCCTGCCACAGGCACAAGTGAGATCGTGGAATATGGTCTCACGGCAAGGGTCGCAATGACCGCTGTGGCAG | 98 | Deletion | 31193 | 3291 | 0.095436 |
| NFXL1-3 | 0 | CAAGGCTGTGGTGGCGGAATAGATGTCCTGCCACAGGCACAAGTGAGATCGTGGAATATGGTCTCACGGCAAGGGTCGCAATGACCGCTGTGGC | 94 | Deletion | 31230 | 1587 | 0.048359 |
| NFXL1-3 | 0 | GCATCTGATATCCTTTGAACCACAAGGGATGTTTCTTAACATCACATGTCCCCCAACACATTCTCTTGTGACTGGCACAAC | 81 | Deletion | 39007 | 195 | 0.004974 |
| NFXL1-3 | 0 | TGCATCTGATATCCTTTGAACCACAAGGGATGTTCCTCAACATCACATGCCCCCCAACACATTCTCTTGTGACTGGCACAACGCAA | 86 | Deletion | 39284 | 3097 | 0.073075 |
| NFXL1-3 | 0 | TGCATCTGATATCCTTTGAACCACAAGGGATGTTCCTCAACATCACATGCCCCCCAACACATTCTCTTGTGACTGGCACAACGCAA | 86 | Deletion | 39401 | 1193 | 0.029389 |
| NFXL1-3 | 1.16E-10 | CATCTGATATCCTTTGAACCACAAGGGATGCTCCTCAACATCACATGCCCCCCAACACATTCTCTTGTGACTGGCACAACGCAA | 84 | Deletion | 38886 | 56 | 0.001438 |
| NFXL1-4 | 0 | CGCAAGGCTGTGGTGGCGGAATAGATGTCCTGCCACAGGCACAAGTGAGATCGTGGAATATGGTCTCACGGCAAGGGTCGCAATGACCGCTGTGGC | 96 | Deletion | 21141 | 130 | 0.006112 |
| NFXL1-4 | 0 | CGCAAGGCTGTGGTGGCGGAATAGATGTCCTGCCACAGGCACAAGTGAGATCGTGGAATATGGTCTCACGGCAAGGGTCGCAGTGACCGCTGTGGCAGAGATGCTGGCATCCACCGCTGTCTCTA | 125 | Deletion | 21108 | 1181 | 0.052986 |
| NFXL1-4 | 0 | CAAGGCTGTGGTGGCGGAATAGATGTCCTGCCACAGGCACAAGTGAGATCGTGGAATATGGTCTCACGGCAAGGGTCGCAATGACCGCTGTGGC | 94 | Deletion | 21134 | 597 | 0.027472 |
| NFXL1-4 | 0 | GATATCCTTTGAACCACAAGGGATGTTCCTCAACATCACATGCCCCCCAACACATTCTCTTGTGACTGGCACAACGCAA | 79 | Deletion | 27826 | 966 | 0.033551 |
| NFXL1-4 | 0 | ATCCTTTGAACCACAAGGGATGTTCCTCAACATCACATGCCCCCCAACACATTCTCTTGTGACTGGCACAACGCAA | 76 | Deletion | 27914 | 523 | 0.018392 |

**D**

| Sample | p-value | Modified sequence (Deleted/ Inserted) | Length (bp) | Editing type | Mapped reads | Reads with modification | Frequency |
| --- | --- | --- | --- | --- | --- | --- | --- |
| pcoNFXL1-1 | 0 | CGCAAGGCTGTGGTGGCGGAATAGATGTCCTGCCACAGGCACAAGTGAGATCGTGGAATATGGTCTCACGGCAAGGGTCGCAATGACCGCTGTGGC | 96 | Deletion | 23577 | 255 | 0.0107 |
| pcoNFXL1-1 | 0 | CAAGGCTGTGGTGGCGGAATAGATGTCCTGCCACAGGCACAAGTGAGATCGTGGAATATGGTCTCACGGCAAGGGTCGCAATGACCGCTGTGGC | 94 | Deletion | 23609 | 491 | 0.020373 |
| pcoNFXL1-1 | 0 | TGCATCTGATATCCTTCGAACCACAGGGGATGTTCCTTAGCATCACATGCCCTCCAACGCACTCTCTCATGACTGGCACAACGCAA | 86 | Deletion | 29071 | 472 | 0.015977 |
| pcoNFXL1-1 | 0 | TGCATCTGATATCCTTTGAACCACAAAGGATGTTCCTCAACATCACATGCCCCCCAACACATTCTCTTGTGACTGGCACAACGCAA | 86 | Deletion | 29082 | 384 | 0.013032 |
| pcoNFXL1-2 | 0 | GCAAGGCTGTGGTGGCGGAATAGATGTCCTGCCACAGGCACAAGTGAGATCGTGGAATATGGTCTCACGGCAAGGGTCGCAATGACCGCTGTGG | 94 | Deletion | 32051 | 608 | 0.018617 |
| pcoNFXL1-2 | 0 | CGCAAGGCTGTGGTGGCGGAATAGATGTCCTGCCACAGGCACAAGTGAGATCGTGGAATATGGTCTCACGGCAAGGGTCGCAATGACCGCTGTGGC | 96 | Deletion | 31928 | 218 | 0.006782 |
| pcoNFXL1-2 | 0 | TGCATCTGATATCCTTTGAACCACAAGGGATGTTCCTCAACATCACATGCCCCCCAACACATTCTCTTGTGACTGGCACAAC | 82 | Deletion | 40024 | 381 | 0.00943 |
| pcoNFXL1-2 | 0 | CATCTGATATCCTTTGAACCACAAGGAATGTTCCTCAACATCACATGCCCCCCAACACATTCTCTTGTGACTGGCACAACG | 81 | Deletion | 40440 | 504 | 0.012309 |
| pcoNFXL1-3 | 0 | GCAAGGCTGTGGTGGCGGAATAGATGTCCTGCCACAGGCACAAGTGAGATCGTGGAATATGGTC TCACGGCAAGGGTCGCAATGACCGCTGTGGCAGAGATGCTGG | 106 | Deletion | 17277 | 429 | 0.024229 |
| pcoNFXL1-3 | 0 | CGCAAGGCTGTGGTGGCGGAATAGATGTCCTGCCACAGGCACAAGTGAGATCGTGGAATATGGTCTCACGGCAAGGGTCGCAGTGACCGCTGTGGCAGAG | 100 | Deletion | 17176 | 235 | 0.013497 |
| pcoNFXL1-3 | 0 | CTGTGGTGGCGGAATAGATGTCCTGCCACAGGCACAAGTGAGATCGTGGAATATGGTCTCACGGCAAGGGTCGCAATGACCGCTGTGGC | 89 | Deletion | 17226 | 345 | 0.019635 |
| pcoNFXL1-3 | 0 | TCCTTTGAACCACAAGGGATGTTCCTCAACATCACATGCCCCCCAACACATTCTCTTGTGACTGGCACAACG | 72 | Deletion | 20925 | 726 | 0.033532 |
| pcoNFXL1-4 | 0 | TTTGTGCCGCAAGGCTGTGGCGGCGGAATAGATGTCCTGCCACAGGCACAAGTGAGATCGTGGAATATGGTCTCACGGCAAGGGTCGCAATGACCGCTGTGGC | 105 | Deletion | 28601 | 107 | 0.003727 |
| pcoNFXL1-4 | 0 | GCAAGGCTGCGGTGGCGGAATAGATGACCTGCCACACGCACACGTGAGATCATTGAATATGGTCTCACGGCAAGGGTCGCAGTGACCGCTGTGGC | 95 | Deletion | 29750 | 155 | 0.005183 |
| pcoNFXL1-4 | 8.37E-08 | GCAAGGCTGTGGTGGTGGAATGGATGTCCTGCCACAGGCACAAGTGAGATCATGGAATATGGTCTCACGGCAAGGGTCACAGTGACCGCTGTGGC | 95 | Deletion | 29785 | 16 | 0.000537 |
| pcoNFXL1-4 | 3.66E-15 | GGCAAGGCTGCGGTGGCGGAATAGATGACCTGCCACACGCACACGTGAGATCATTGAATATGGTCTCACGGCAAGGGTCGCAGTGACCGCTGTGG | 95 | Deletion | 29961 | 25 | 0.000834 |
| pcoNFXL1-4 | 0 | CGCAAGGCTGTGGTGGTGGAATGGATGTCCTGCCACAGGCACAAGTGAGATCATGGAATATGGTCTCACGGCAAGGGTCACAGTGACCGCTGTGGC | 96 | Deletion | 29945 | 255 | 0.008444 |
| pcoNFXL1-4 | 0 | CAAGGCTGTGGTGGTGGAATGGATGTCCTGCCACAGGCACAAGTGAGATCATGGAATATGGTCTCACGGCAAGGGTCACAGTGACCGCTGTGGCAGAGATGCTGGCATCCA | 111 | Deletion | 30020 | 265 | 0.00875 |
| pcoNFXL1-4 | 0 | TGCATCTGATATCCTTTGAACCACAAGGGATGTTCCTCAACATCACATGCCCCCCAACACATTCTCTT | 68 | Deletion | 37027 | 776 | 0.020527 |
| pcoNFXL1-4 | 0 | TGCATCTGATATCCTTTGAACCACAAGGGATGTTCCTTAACATCACATGTCCCCCAACGCACTCTCTCATGACTGGCACAACGCAA | 86 | Deletion | 37482 | 101 | 0.002687 |
| pcoNFXL1-4 | 0 | CCTCAACATCACATGCCCCCCAACACATTCTCTTGTGACTGGCACAACGCAA | 52 | Deletion | 37471 | 33 | 0.00088 |
| pcoNFXL1-5 | 0 | CGCAAGGCTGTGGTGGCGGAATAGATGTCCTGCCACAGGCACAAGTGAGATCGTGGAATATGGTCTCACGGCAAGGGTCGCAATGACCGCTGTGGCAGAG | 100 | Deletion | 20455 | 714 | 0.033729 |
| pcoNFXL1-5 | 0 | CGCAAGGCTGTGGTGGCGGAATAGATGTCCTGCCACAGGCACAAGTGAGATCGTGGAATATGGTCTCACGGCAAGGGTCGCAGTGACCGCTGTGGCAGAGATGCTGGCATCCA | 113 | Deletion | 20372 | 1587 | 0.072271 |
| pcoNFXL1-5 | 0 | CAAGGCTGTGGTGGTGGAATGGATGTCCTGCCACAGGCACAAGTGAGATCATGGAATATGGTCTCACGGCAAGGGTCACAGTGACCGCTGTGGC | 94 | Deletion | 20421 | 189 | 0.00917 |
| pcoNFXL1-5 | 0 | TGCATCTGATATCCTTTGAACCACAAGGGATGTTCCTCAACATCACATGCCCCCCAACACATTCTCTTGTGACTGGCACAAC | 82 | Deletion | 25164 | 1120 | 0.042611 |
| pcoNFXL1-5 | 0 | TGCATCTGATATCCTTTGAACCACAAGGGATGTTCCTCAACATCACATGCCCCCCAACACATTCTCTTGTGACTGGCACAACGCAA | 86 | Deletion | 25421 | 1325 | 0.04954 |

E

| Sample | Deleted sequence | Length (bp) | Frequency |
| --- | --- | --- | --- |
| ABCC6-1 | CTGGAGGACGAGAACTCTGAATTCTCCAGGCTCATCAAGGAGTACTCACGGAGATC | 56 | 0.077099 |
| ABCC6-2 | CTGGAGGACGAGAACTCTGAATTCTCCAGGCTCATCAAGGAGTACTCACGGAGATC | 56 | 0.064906 |
| ABCC6-3 | CTGGAGGACGAGAACTCTGAATTCTCCAGGCTCATCAAGGAGTACTCACGGAGATC | 56 | 0.084866 |
| ABCC6-4 | CTGGAGGACGAGAACTCTGAATTCTCCAGGCTCATCAAGGAGTACTCACGGAGATC | 56 | 0.097029 |
| ABCC6-5 | CTGGAGGACGAGAACTCTGAATTCTCCAGGCTCATCAAGGAGTACTCACGGAGATC | 56 | 0.086561 |
| nsLTPC6-1 | GTGACGGGGAGGGCGTCCTCGATCAGCAAGGAGTGCTGCTCCGGCGT | 47 | 0.086013 |
| nsLTP9.4-2 | GTGACGGGGAGGGCGTCCTCGATCAGCAAGGAGTGCTGCTCCGGCGT | 47 | 0.008538 |
| nsLTP9.4-2 | GTGACGGGGAGGGCGTCCTCGATCAGCAAGGAGTGCTGCTCCGGCGTG | 48 | 0.008501 |
| nsLTP9.4-3 | GTGACGGGGAGGGCGTCCTCGATCAGCAAGGAGTGCTGCTCCGGCGT | 47 | 0.095866 |
| nsLTP9.4-4 | GTGACGGGGAGGGCGTCCTCGATCAGCAAGGAGTGCTGCTCCGGCGT | 47 | 0.099019 |
| pcoNFXL1-4 | GTGACTGGCACAACGCAAGGTGGGCAGTCCCCGAAATGGCATTGATGCGAAGCTGGATGCCCGCAAGGCTGAGGGACCATGCACTGRTGTGGGCATGATGGAGTTGG | 107 | 0.041357 |

**F**

| Sample | Original sequence | Substitution sequence | Frequency |
| --- | --- | --- | --- |
| NFXL1-1 | GGTGGGCAGTCCCCGAAATGGCATTGATGCGAAGCTGGATGCCCGCAAGGCTGAGGGACCATGCACTGRTGTGGGCATGATGGAGTTGGTGTGCCGCAAGGCTGTGGTGGCGGAATAGATGTCCTGCCACAGGCACAAGTGAGATCR | CGCAAGGCTGTGGTGGCGGAATAGATGTCCTGCCACAGGCACAAGTGAGATCG | 0.039408 |
| NFXL1-2 | GGTGGGCAGTCCCCGAAATGGCATTGATGCGAAGCTGGATGCCCGCAAGGCTGAGGGACCATGCACTGRTGTGGGCATGATGGAGTTGGTGTGCCGCAA | CGCAAGGCTGCGGTGGCGGAATAGATGACCTGCCACACGCACAAGAGAGATCATTGAATATGGTCTCACGGCAAGGGTCGCAGTGACCGCTGTGGCAGAGATGCTGGCATCCACCTGTCTCTTATAAGAGGACAGAGCTTTCCACAGGGCTGGTTGCATCTGATATCCTTTGAACCACAGGGGATGTTCCTTAGCATCACATGTCCTCCAACGCACTCTCTCATGACTGGCACAACGCAAGCAA | 0.072017 |
| NFXL1-3 | GGTGGGCAGTCCCCGAAATGGCATTGATGCGAAGCTGGATGCCCGCAAGGCTGAGGGACCATGCACTGRTGTGGGCATGATGGAGTTGGTGTGCCGCAAGGCTGTGGTGGCGGAATAGATGTCCTGCCACAGGCACAAGTGAGATCR | CGCAAGGCTGTGGTGGCGGAATAGATGTCCTGCCACAGGCACAAGTGAGATCG | 0.145537 |
| NFXL1-4 | GGTGGGCAGTCCCCGAAATGGCATTGATGCGAAGCTGGATGCCCGCAAGGCTGAGGGACCATGCACTGRTGTGGGCATGATGGAGTTGGTGTGCCGCAAGGCTGTGGTGGCGGAATAGATGTCCTGCCACAGGCACAAGTGAGATCRTGGAATATGGTCTCACGGCAAGGGTCGCAATGACCGCTGTGGCAGAGATGCTGGCATCCA | CGCAAGGCTGTGGTGGCGGAATAGATGTCCTGCCACAGGCACAAGTGAGATCGTGGAATATGGTCTCACGGCAAGGGTCGCAGTGACCGCTGTGGCAGAGATGCTGGCATCCACCGCTGTCTCTA | 0.089956 |
| pcoNFXL1-1 | GGTGGGCAGTCCCCGAAATGGCATTGATGCGAAGCTGGATGCCCGCAAGGCTGAGGGACCATGCACTGRTGTGGGCATGATGGAGTTGGTGTGCCGCAAGGCTGTGGTGGCGGAATAGATGTCCTGCCACAGGCACAAGTGAGATCR | CGCAAGGCTGTGGTGGCGGAATAGATGTCCTGCCACAGGCACAAGTGAGATCG | 0.042286 |
| pcoNFXL1-2 | AAGGTGGGCAGTCCCCGAAATGGCATTGATGCGAAGCTGGATGCCCGCAAGGCTGAGGGACCATGCACTGRTGTGGGCATGATGGAGTTGGTGTGCCGCAAGGCTGTGGTGGCGGAATAGATGTCCTGCCACAGGCACAAGTGAGATCR | GCAAGGCTGTGGTGGCGGAATAGATGTCCTGCCACAGGCACAAGTGAGATCG | 0.038925 |
| pcoNFXL1-3 | AAGGTGGGCAGTCCCCGAAATGGCATTGATGCGAAGCTGGATGCCCGCAAGGCTGAGGGACCATGCACTGRTGTGGGCATGATGGAGTTGGTGTGCCGCAAGGCTGTGGTGGCGGAATAGATGTCCTGCCACAGGCACAAGTGAGATCR | GCAAGGCTGTGGTGGCGGAATAGATGTCCTGCCACAGGCACAAGTGAGATCG | 0.059226 |
| pcoNFXL1-4 | GGTGGGCAGTCCCCGAAATGGCATTGATGCGAAGCTGGATGCCCGCAAGGCTGAGGGACCATGCACTGRTGTGGGCATGATGGAGTTGGTGTGCCGCAAGGCTGTGGTGGCGGAATAGATGTCCTGCCACAGGCACAAGTGAGATCRTGGAATATGGTCTCACGGCAAGGGTCGCAATGACCGCTGTGGCAGAGATGCTGGCATCCA | CGCAAGGCTGTGGTGGCGGAATAGATGTCCTGCCACAGGCACAAGTGAGATCGTGGAATATGGTCTCACGGCAAGGGTCGCAGTGACCGCTGTGGCAGAGATGCTGGCATCCAACTGTCTCTTATAC | 0.118976 |
