# Supplementary material for: An optimised CRISPR/Cas9 protocol to create targeted mutations in homoeologous genes and an efficient genotyping protocol to identify edited events in wheat
Source: Plant Methods. 2019 Oct 24;15:119. doi: 10.1186/s13007-019-0500-2 (PMC6814032; doi:10.1186/s13007-019-0500-2)
Supplement: Supplementary file 8 — Additional file 8. Estimated editing efficiency in the protoplast system at the individual target sites for sgRNA-1 and sgRNA-2. [file 13007_2019_500_MOESM8_ESM.docx]

**Additional file 8.** Estimated editing efficiency in the protoplast system at the individual target sites for sgRNA-1 and sgRNA-2.

| Sample | Editing efficiency^a^ | | Sample | Editing efficiency | |
| --- | --- | --- | --- | --- | --- |
|  | sgRNA-1 | sgRNA-2 |  | sgRNA-1 | sgRNA-2 |
| ABCC6-1 | 8.4% | 8.6% | NFXL1-1 | 1.8% | 6.6% |
| ABCC6-2 | 6.6% | 6.5% | NFXL1-2 | 11.7% | 13.8% |
| ABCC6-3 | 8.8% | 8.8% | NFXL1-3 | 10.9% | 31.3% |
| ABCC6-4 | 11.1% | 11.6% | NFXL1-4 | 5.2% | 17.7% |
| ABCC6-5 | 8.7% | 8.9% | NFXL1-5 | 0.0%^b^ | 0.0%^b^ |
| nsLTP9.4-1 | 9.1% | 8.6% | pcoNFXL1-1 | 2.9% | 7.3% |
| nsLTP9.4-2 | 1.9% | 1.7% | pcoNFXL1-2 | 2.2% | 6.4% |
| nsLTP9.4-3 | 11.3% | 9.6% | pcoNFXL1-3 | 3.4% | 11.7% |
| nsLTP9.4-4 | 11.9% | 9.9% | pcoNFXL1-4 | 6.5% | 18.8% |
| nsLTP9.4-5 | 0.0%^b^ | 0.0%^b^ | pcoNFXL1-5 | 9.2% | 11.5% |

^a^ For modifications between two sgRNA target sites, they were counted in both categories.

^b^No modification was detected at this sgRNA target site.
