# Supplementary material for: An optimised CRISPR/Cas9 protocol to create targeted mutations in homoeologous genes and an efficient genotyping protocol to identify edited events in wheat
Source: Plant Methods. 2019 Oct 24;15:119. doi: 10.1186/s13007-019-0500-2 (PMC6814032; doi:10.1186/s13007-019-0500-2)
Supplement: Supplementary file 9 — Additional file 9. Vector used for wheat transformation. (A) Cloning strategy using the Gateway recombination system to introduce two fragments into the vector pVB29. (B) Final vector used for wheat microprojectile bombardment. [file 13007_2019_500_MOESM9_ESM.pptx]

## Slide 1
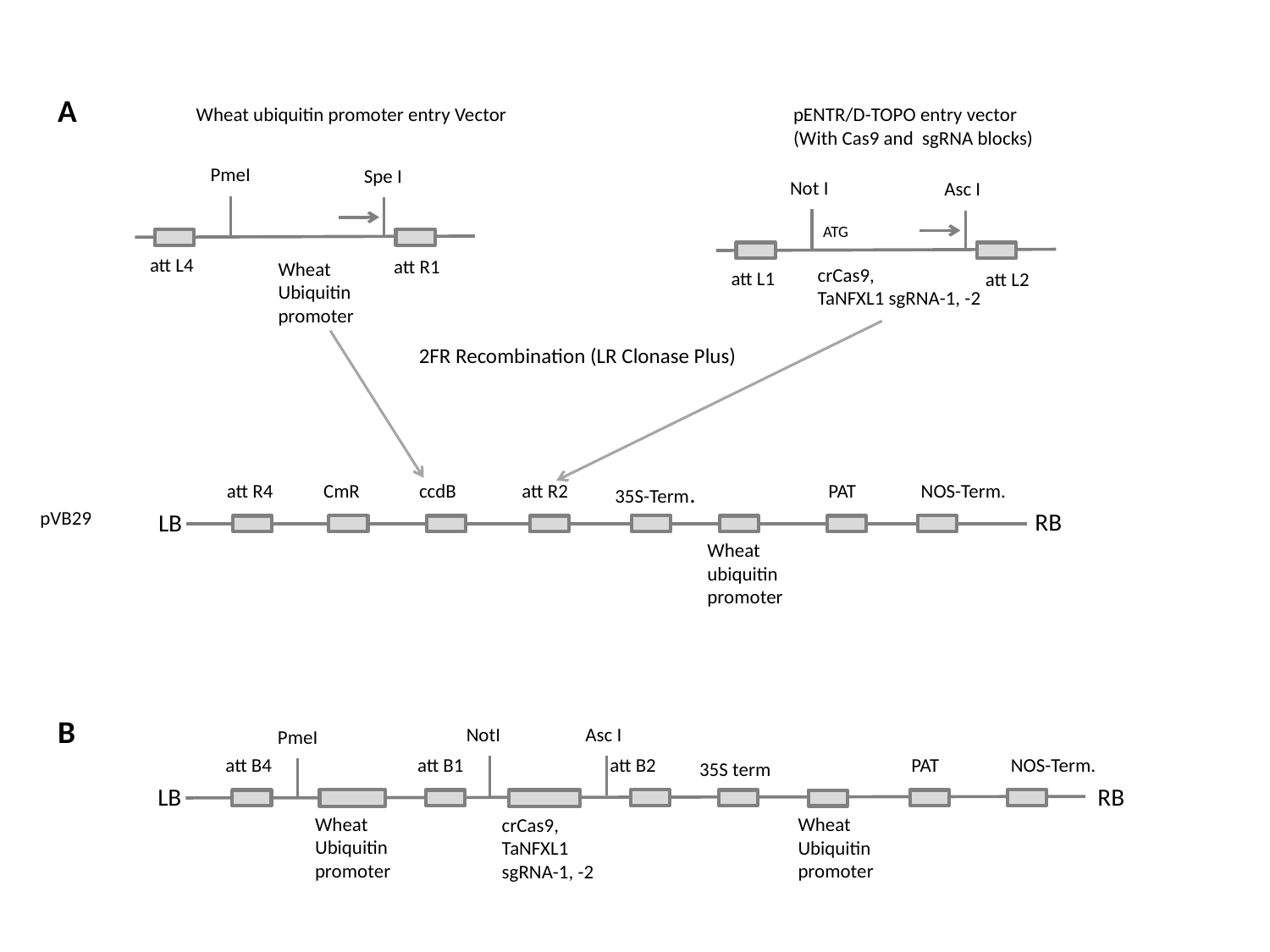

A
Wheat ubiquitin promoter entry Vector
pENTR/D-TOPO entry vector
(With Cas9 and sgRNA blocks)
PmeI
Spe I
Not I
Asc I
ATG
att L4
att R1
Wheat Ubiquitin
promoter
crCas9,
TaNFXL1 sgRNA-1, -2
att L1
att L2
2FR Recombination (LR Clonase Plus)
att R4
CmR
ccdB
att R2
35S-Term.
PAT
NOS-Term.
RB
LB
Wheat
ubiquitin
promoter
pVB29
B
NotI
Asc I
PmeI
att B1
att B4
att B2
PAT
NOS-Term.
35S term
RB
LB
Wheat Ubiquitin
promoter
Wheat
Ubiquitin
promoter
crCas9,
TaNFXL1 sgRNA-1, -2
