# Supplementary material for: An optimised CRISPR/Cas9 protocol to create targeted mutations in homoeologous genes and an efficient genotyping protocol to identify edited events in wheat
Source: Plant Methods. 2019 Oct 24;15:119. doi: 10.1186/s13007-019-0500-2 (PMC6814032; doi:10.1186/s13007-019-0500-2)
Supplement: Supplementary file 11 — Additional file 11. Sequence of the primers used in this study. [file 13007_2019_500_MOESM11_ESM.docx]

**Additional file 11.** Sequence of the primers used in this study.

| Primer name | Sequence (5'-3') | Purpose |  |
| --- | --- | --- | --- |
| ABC2-F3398-P2 | CGGACAGAGGCAGTTGTTT | sgRNA *in vitro* test |  |
| ABC2-R3913-P2 | GAGAAAGGTCGTTGTGGTGT |  |  |
| nsLTP-F77-P2 | CACACTCATCTGATCACCCATAG |  |  |
| nsLTP-R526-P2 | TCAGGAGACCGAAAGGTTAGA |  |  |
| NFXL1-F314-P1 | CAGATGGTGGAGTGCTACAA |  |  |
| NFXL1-R1069-P1 | GTAGTGATACGGCCACAAGAA |  |  |
| Gib_assem_EcoRI-1F | CGGAATTCCGCGGTGTCATCTATGTTAC^a^ | Cloning into pCambia 1302 |  |
| Gib_assem_KpnI_1052R | GGGGTACCACCCGCCAATATATCCTGTC |  |  |
| Gib_XmaI_R | TCCCCCCGGGACCCGCCAATATATCCTGTC |  |  |
| Cas9/sgRNA block_NotI_F | AGTTGCGGCCGCATGGACAAGAAGTACAGCATCGGCCTGGAC^b^ | Cloning into pENTR/D-TOP |  |
| Cas9/sgRNA block _AscI_R | TTGCGGCGCGCCCAATATATCCTGTCAAACACTGATAGTTTAATTGGTAC |  |  |
| pJet_Seq_F  pJet_Seq_R | CGACTCACTATAGGGAGAGCGGC  AAGAACATCGATTTTCCATGGCAG | Sanger sequencing after cloning into pJet1.2 |  |
| Gib_assem_1F | CGCGGTGTCATCTATGTTAC | Sanger sequencing after cloning into expression vectors |  |
| Gib_assem_1052R | ACCCGCCAATATATCCTGTC |  |  |
| nsLTP-F77-P2 | CACACTCATCTGATCACCCATAG | Gene-specific primers for HTS |  |
| nsLTP-R526-P2 | TCAGGAGACCGAAAGGTTAGA |  |  |
| Illu_Rd1_0N_ABC-F | *TCGTCGGCAGCGTCAGATGTGTATAAGAGACAG*TGTCTTCAGCGAAGGTAAGC^c^ | Primers with barcode for HTS |  |
| Illu_Rd1_1N_ABC-F | *TCGTCGGCAGCGTCAGATGTGTATAAGAGACAGN*TGTCTTCAGCGAAGGTAAGC |  |  |
| Illu_Rd1_2N_ABC-F | *TCGTCGGCAGCGTCAGATGTGTATAAGAGACAGNN*TGTCTTCAGCGAAGGTAAGC |  |  |
| Illu_Rd1_3N_ABC-F | *TCGTCGGCAGCGTCAGATGTGTATAAGAGACAGNNN*TGTCTTCAGCGAAGGTAAGC |  |  |
| Illu_Rd1_0N_ABC-R | *GTCTCGTGGGCTCGGAGATGTGTATAAGAGACAG*GGTGTGTGCGGTCTTTCT |  |  |
| Illu_Rd1_1N_ABC-R | *GTCTCGTGGGCTCGGAGATGTGTATAAGAGACAGN*GGTGTGTGCGGTCTTTCT |  |  |
| Illu_Rd1_2N_ABC-R | *GTCTCGTGGGCTCGGAGATGTGTATAAGAGACAGNN*GGTGTGTGCGGTCTTTCT |  |  |
| Illu_Rd1_3N_ABC-R | *GTCTCGTGGGCTCGGAGATGTGTATAAGAGACAGNNN*GGTGTGTGCGGTCTTTCT |  |  |
| Illu_Rd1_0N_NFXL-F | *TCGTCGGCAGCGTCAGATGTGTATAAGAGACAG*CTTTCCACAGGGCTGGTT |  |  |
| Illu_Rd1_1N_NFXL-F | *TCGTCGGCAGCGTCAGATGTGTATAAGAGACAGN*CTTTCCACAGGGCTGGTT |  |  |
| Illu_Rd1_2N_NFXL-F | *TCGTCGGCAGCGTCAGATGTGTATAAGAGACAGNN*CTTTCCACAGGGCTGGTT |  |  |
| Illu_Rd1_3N_NFXL-F | *TCGTCGGCAGCGTCAGATGTGTATAAGAGACAGNNN*CTTTCCACAGGGCTGGTT |  |  |
| Illu_Rd1_0N_NFXL-R | *GTCTCGTGGGCTCGGAGATGTGTATAAGAGACAG*TGGATGCCAGCATCTCTG |  |  |
| Illu_Rd1_1N_NFXL-R | *GTCTCGTGGGCTCGGAGATGTGTATAAGAGACAGN*TGGATGCCAGCATCTCTG |  |  |
| Illu_Rd1_2N_NFXL-R | *GTCTCGTGGGCTCGGAGATGTGTATAAGAGACAGNN*TGGATGCCAGCATCTCTG |  |  |
| Illu_Rd1_3N_NFXL-R | *GTCTCGTGGGCTCGGAGATGTGTATAAGAGACAGNNN*TGGATGCCAGCATCTCTG |  |  |
| NFXL1_AX_F | CAGTCGGGCGTCATCACAC GGAGTGCTACAAGGTCTCGAT | Genotyping |  |
| NFXL1_AX_R | AGCTTCTCCATTTGCAGGTGGC |  |  |
| NFXL1_AY_F | CAGTCGGGCGTCATCACAC TTGTGGCCGCAAGAAGAACTGT |  |  |
| NFXL1_AY_R | GRGTGGTGTCCCAAGGAGG |  |  |
| NFXL1_BX_F | CAGTCGGGCGTCATCACAC CTCTGTTGCCCTCTGTCAAAGCC |  |  |
| NFXL1_BX_R | CTTGGGACACCACATAACTGC |  |  |
| NFXL1_BY_F | CAGTCGGGCGTCATCACAC GAAGAACTGCGGGAGGCACC |  |  |
| NFXL1_BY_R | GAAACTTTGCCCCCAGAGCC |  |  |
| NFXL1_DX_F | CAGTCGGGCGTCATCACAC GATGGTGGAGTGCTACAAGGTATT |  |  |
| NFXL1_DX_R | TGACGACGGGTGGCATGGC |  |  |
| NFXL1_DY_F | CAGTCGGGCGTCATCACAC GCCATTTGCTCAGCATGAAGGC |  |  |
| NFXL1_DY_R | ATGTTCACATCTCACGTCTGGA |  |  |
| Universal primer | CAGTCGGGCGTCATCACAC^d^ |  |  |

^a^Underlined bases indicate EcoRI, Kpn1 and XmaI restriction sites.

^b^Underlined bases indicate NotI and AscI restriction sites

^c^Adaptor sequences are in italic while the gene-specific primers are in red.

^d^FAM-, NED-, VIC-fluorescence-labeled versions were used for subgenome A, B and D respectively.
