# Supplementary material for: An optimised CRISPR/Cas9 protocol to create targeted mutations in homoeologous genes and an efficient genotyping protocol to identify edited events in wheat
Source: Plant Methods. 2019 Oct 24;15:119. doi: 10.1186/s13007-019-0500-2 (PMC6814032; doi:10.1186/s13007-019-0500-2)
Supplement: Supplementary file 12 — Additional file 12. gBlock components. (A) Schematic representation of components in a gBlock. Overlap: sequences used for Gibson assembly or cloning; U6 promoter: wheat U6 promoter [32] for transcription of sgRNA and sgRNA scaffold. (B) Full sequences for two gBlocks (gBlocks 1 and 2; 546 bp for one block). In italic: 40 bp overlap with the pCambia vector; underlined: wheat U6 promoter; 20 Ns in blue: position of sgRNA sequence; in green: sgRNA scaffold and terminator; in red: 40 bp overlapping sequence between the two gBlocks for Gibson assembly [34]. [file 13007_2019_500_MOESM12_ESM.docx]

**A**


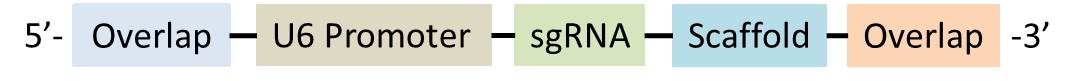


**B**

gBlock1: *CGCGGTGTCATCTATGTTACTAGATCGGGGAGCTCGAATT*GACCAAGCCCGTT ATTCTGACAGTTCTGGTGCTCAACACATTTATATTTATCAAGGAGCACATTGTTACTCACTGCTAGGAGGGAATCGAACTAGGAATATTGATCAGAGGAACTACGAGAGAGCTGAAGATAACTGCCCTCTAGCTCTCACTGATCTGGGTCGCATAGTGAGATGCAGCCCACGTGAGTTCAGCAACGGTCTAGCGCTGGGCTTTTAGGCCCGCATGATCGGGCTTTTGTCGGGTGGTCGACGTGTTCACGATTGGGGAGAGCAACGCAGCAGTTCCTCTTAGTTTAGTCCCACCTCGCCTGTCCAGCAGAGTTCTGACCGGTTTATAAACTCGCTTGCTGCATCAGACTTGNNNNNNNNNNNNNNNNNNNNGTTTTAGAGCTAGAAATAGCAAGTTAAAATAAGGCTAGTCCGTTATCAACTTGAAAAAGTGGCACCGAGTCGGTGCTTTTTTTAGATGGGGCGACGAGTTACTGGCCCTGATTTCTCCGCTTC

gBlock2: AGATGGGGCGACGAGTTACTGGCCCTGATTTCTCCGCTTCGACCAAGCCCG TTATTCTGACAGTTCTGGTGCTCAACACATTTATATTTATCAAGGAGCACATTGTTACTCACTGCTAGGAGGGAATCGAACTAGGAATATTGATCAGAGGAACTACGAGAGAGCTGAAGATAACTGCCCTCTAGCTCTCACTGATCTGGGTCGCATAGTGAGATGCAGCCCACGTGAGTTCAGCAACGGTCTAGCGCTGGGCTTTTAGGCCCGCATGATCGGGCTTTTGTCGGGTGGTCGACGTGTTCACGATTGGGGAGAGCAACGCAGCAGTTCCTCTTAGTTTAGTCCCACCTCGCCTGTCCAGCAGAGTTCTGACCGGTTTATAAACTCGCTTGCTGCATCAGACTTGNNNNNNNNNNNNNNNNNNNNGTTTTAGAGCTAGAAATAGCAAGTTAAAATAAGGCTAGTCCGTTATCAACTTGAAAAAGTGGCACCGAGTCGGTGCTTTTTTT*CAATTAAACTATCAGTGTTTGACAGGATATATTGGCGGGT*
